# Informed walks: whispering hints to gene hunters inside networks’ jungle

**DOI:** 10.1186/s12918-017-0473-6

**Published:** 2017-10-11

**Authors:** Marilena M. Bourdakou, George M. Spyrou

**Affiliations:** 1 Bioinformatics ERA Chair, The Cyprus Institute of Neurology and Genetics, 6 International Airport Avenue, Ayios Dometios, 2370 Nicosia, Cyprus; 2 Center of Systems Biology, Biomedical Research Foundation, Academy of Athens, Soranou Ephessiou 4, 115 27 Athens, Greece

**Keywords:** Random walks, Network inference, Network analysis, Gene subnetworks, Molecular mechanisms, Drug repurposing, Cancer types

## Abstract

**Background:**

Systemic approaches offer a different point of view on the analysis of several types of molecular associations as well as on the identification of specific gene communities in several cancer types. However, due to lack of sufficient data needed to construct networks based on experimental evidence, statistical gene co-expression networks are widely used instead. Many efforts have been made to exploit the information hidden in these networks. However, these approaches still need to capitalize comprehensively the prior knowledge encrypted into molecular pathway associations and improve their efficiency regarding the discovery of both exclusive subnetworks as candidate biomarkers and conserved subnetworks that may uncover common origins of several cancer types.

**Methods:**

In this study we present the development of the *Informed Walks* model based on random walks that incorporate information from molecular pathways to mine candidate genes and gene-gene links. The proposed model has been applied to TCGA (The Cancer Genome Atlas) datasets from seven different cancer types, exploring the reconstructed co-expression networks of the whole set of genes and driving to highlighted sub-networks for each cancer type. In the sequel, we elucidated the impact of each subnetwork on the indication of underlying exclusive and common molecular mechanisms as well as on the short-listing of drugs that have the potential to suppress the corresponding cancer type through a drug-repurposing pipeline.

**Conclusions:**

We have developed a method of gene subnetwork highlighting based on prior knowledge, capable to give fruitful insights regarding the underlying molecular mechanisms and valuable input to drug-repurposing pipelines for a variety of cancer types.

**Electronic supplementary material:**

The online version of this article (10.1186/s12918-017-0473-6) contains supplementary material, which is available to authorized users.

## Background

Some of the most devastating forms of cancer have genetic similarities, even though they appear to different tissues and organs of the body. For example, one type of breast cancer presents genetic mutations very similar to the ones found in ovarian cancer, while colon cancers often have mutations found in breast cancer. Also, according to several studies, the most aggressive form of endometrial cancer affecting the uterine lining is similar to more grave forms of breast and ovarian cancer [1, 2]. Such similarities between different cancer types make our understanding on them a challenging and fascinating task.

Despite the rapid increase in human cancer-associated gene discovery, a large proportion of specific cancer-associated genes is still unknown [3]. Network-based approaches support the study of interactions among relatively large number of genes, aiming to propose lists of statistically significant genes for each human cancer type [4]. The majority of these approaches utilize the protein-protein interaction (PPI) network or prior knowledge to highlight significant genes. However, due to the lack of functional characterization for a significant number of genes, these approaches are not as informative as expected. In order to overcome this limitation, many network inference methods have been adopted to reconstruct co-expression networks based on gene expression data regarding certain diseases [5–8].

So far, many methods have been employed for disease-specific gene-mining, using molecular networks to perform local and global distance measurements, clustering methods, diffusion kernels and random walks with restarts [9–12]. Among these, random walks have shown promising results in prioritizing disease genes, finding distances between correlated nodes and highlighting genes related to specific diseases [13, 14]. However, these methods are limited because they examine only the topology of the networks and the weights of the edges without including functional information of genes.

In this study, we developed a method called Informed Walks, based on random walk theory with restarts by incorporating information from molecular pathways, in order to mine important genes and gene-gene links related to seven different cancer types. Specifically, we have used all the available mRNA gene expression microarray datasets retrieved from The Cancer Genome Atlas – TCGA (http://gdac.broadinstitute.org/runs/stddata__latest/samples_report/) and we have reconstructed co-expression networks for seven different cancer types using the whole set of genes. We applied the Informed Walks model in the co-expression networks and we concluded to a highlighted sub-network for each cancer type. Analyzing each sub-network, we identified specific mechanisms significant for each cancer type while the significant genes derived from each sub-network were used in a drug repurposing pipeline, revealing drugs that have the potential to suppress each cancer type. Finally, common and exclusive mechanisms as well as the impact of the repurposed drugs were investigated across the different cancer types.

## Results

**Fig. 1:**
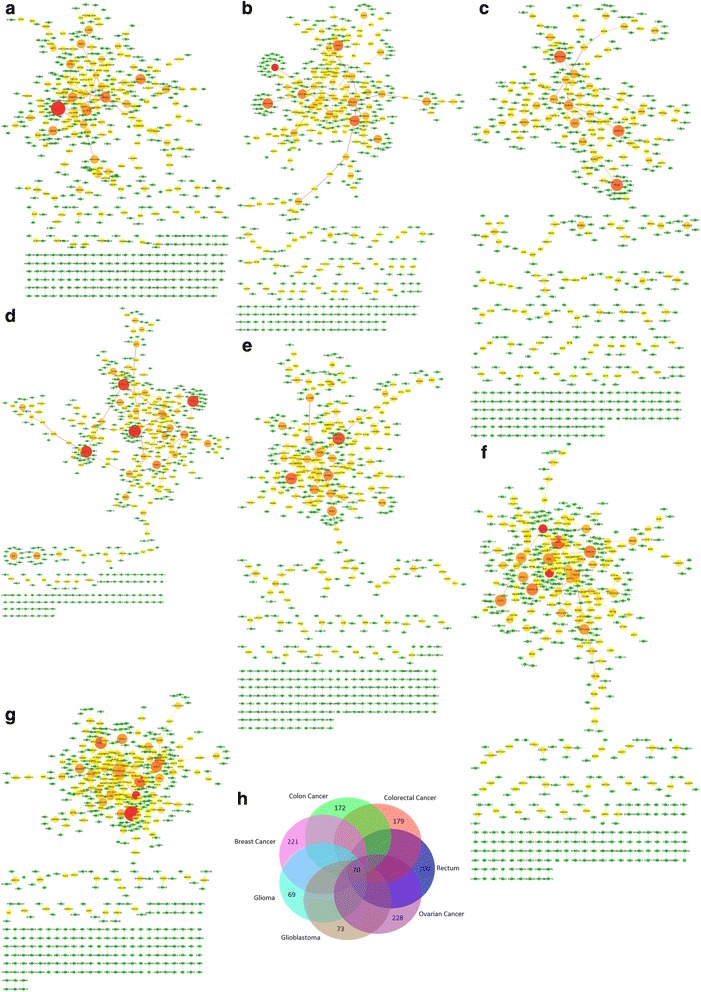
Top 500 edges for the subnetworks of each cancer type. The node size and color correspond to the degree centrality (higher values are represented by bigger and darker nodes). The edge size and color correspond to the edge betweenness (higher values are represented by bigger and darker edges). **a**) Breast cancer subnetwork **b**) colon cancer subnetwork **c**) colorectal cancer subnetwork **d**) rectum subnetwork **e**) ovarian cancer subnetwork **f**) glioblastoma subnetwork **g**) glioma subnetwork **h**) representation of common and exclusive genes between the seven subnetworks

The networks identified for each cancer type, as generated by merging each walker’s pathways, have edges with weights that are proportional to the frequency of passage through the corresponding edge. To simplify the resulted subnetworks, the edges with the top 500 higher weight values were retained for each cancer type highlighting subnetworks with 574 genes-nodes in the case of breast cancer (Fig. 1a), 547 genes-nodes in the case of colon cancer (Fig. 1b), 593 genes-nodes in the case of colorectal cancer (Fig. 1c), 523 genes-nodes in the case of rectum (Fig. 1d), 585 genes-nodes in the case of ovarian cancer (Fig. 1e), 544 genes-nodes in the case of glioblastoma (Fig. 1f) and 536 genes-nodes in the case of glioma (Fig. 1g). Common and exclusive genes for each cancer type were further investigated (Fig. 1h). The overlap between the seven subnetworks is available in Additional file 1: Table S1.

In total, 70 common genes were identified among the seven cancer types. By using the Enrichr web-based software (http://amp.pharm.mssm.edu/Enrichr/) [15], we found that these genes were involved in several important mechanisms for carcinogenesis and cancer progression. More specifically, 17 out of 70 genes (HIST1H4K, HIST1H4L, MAOA, GNG12, HIST1H4A, NRAS, HIST1H4B, HIST1H4H, CALM3, HIST1H4J, HIST1H4C, CALM1, CALM2, HIST1H4D, HRAS, HIST1H4E and HIST1H4F) are involved in alcoholism pathway. Based on extensive reviews of research studies, there is a strong scientific consensus of an association between alcohol drinking and several types of cancer [16, 17]. Furthermore 16 out of 70 genes are involved in viral carcinogenesis pathway (CDKN1A, CDKN1B, HIST1H4K, HIST1H4L, HIST1H4A, NRAS, HIST1H4B, CASP3, HIST1H4H, HIST1H4J, HIST1H4C, HIST1H4D, TP53, HRAS, HIST1H4E and HIST1H4F) and 17 in pathways in cancer (CDKN1A, CDKN1B, TGFB1, EGF, ADCY8, GNG12, NRAS, FGF9, AKT2, CASP3, AKT1, PLCB1, BID, WNT1, TP53, HRAS and FGF23).

We have found 221 exclusive genes related to breast cancer. The most important mechanism based on these genes was found to be hepatitis b. HBV was positively associated with breast cancer as patients undergoing chemotherapy for breast cancer have higher rate of HBV reactivation than other cancer patients [18]. As HBV is inactive in these patients, this association may reflect an immune response signature.

We have also revealed 172 exclusive genes for colon cancer and 179 for colorectal cancer. One of the most important pathways that were found based on the exclusive genes of colon and colorectal cancer is the pathway of proteoglycans in cancer. It has been found that proteoglycans may play a pivotal role as potential microenvironmental biomarkers for colon cancer diagnostics and treatment [19]. Furthermore, our analysis highlights 200 exclusive genes for rectum and 228 for the ovarian cancer. RNA transport and spliceosome were found to be important mechanisms in the case of rectum whereas proteasome and ether lipid metabolism were highlighted respectively in the case of ovarian cancer. It has been reported that some lipid metabolic enzymes are overexpressed in ovarian cancer [20].

Finally, 73 and 69 exclusive genes were found for glioblastoma and glioma respectively. The most important molecular mechanism that was found from the 73 exclusive genes of glioblastoma is the antigen processing and presentation pathway. Alterations of this pathway have been found in glioblastoma [21]. Fc gamma R-mediated phagocytosis and adipocytokine signaling pathway were found as the most important pathways from the exclusive genes in the case of glioma.

The significant pathways from the common and exclusive genes of the seven cancer types are presented in Additional file 1: Table S2, Table S3, Table S4, Table S5, Table S6, Table S7, Table S8 and Table S9.

### Underlying mechanism discovery

**Fig. 2:**
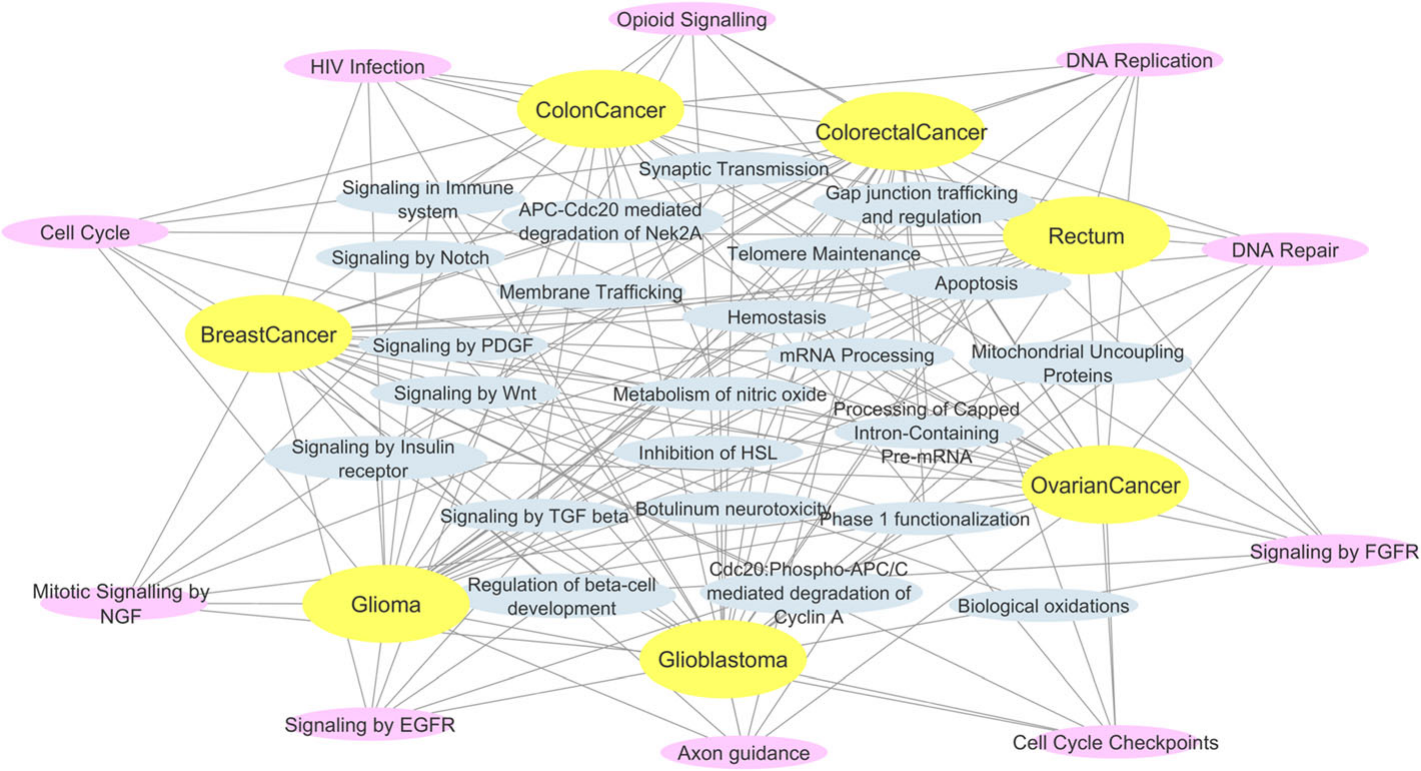
Pathway – Disease Network. Nodes with blue color represent the significant mechanisms of each cancer type, nodes with yellow color represent the seven different cancer types and with pink color the 10 common mechanisms between all cancer types

We used the Enrichr web-based software in order to reveal the underlying significant biological pathways derived from the genes of each sub-network. From the seven cancer types we highlighted the significant pathways (*p*-value <0.05) (Additional file 1: Table S10, Table S11, Table S12, Table S13, Table S14, Table S15 and Table S16). Common and exclusive mechanisms of each cancer type were further investigated (Additional file 1: Table S17). Following pathway analysis of the seven cancer types, we have found ten common cancer – related pathways (Fig. 2) such as axon guidance, cell cycle checkpoints, signaling by FGFR, DNA repair, DNA replication, opioid signaling, HIV infection, cell cycle, mitotic signaling by NGF, and signaling by EGFR. DNA repair processes and cell cycle checkpoints have been intimately linked with cancer due to their functions regulating genome stability and cell progression, respectively. Furthermore, cancer and mitosis are closely related to each other. Without the process of mitosis there would be no cancer. Mitosis is the process by which cells reproduce. Without mitosis cancerous cells wouldn’t be able to form tumors and spread through the body. Mistakes that occur during DNA replication can lead to the generation of cells with mutated genes. Accumulations of mutations can lead to the development of cancer. There are several cancer types that are associated specifically with the breakdown of the repair processes that normally function during DNA replication. Moreover it has been reported, that axon guidance pathway plays a pivotal role in tumorigenesis [22, 23]. It has been also reported that aberrant FGFR signaling contributes to carcinogenesis [24]. Opioids promote angiogenesis, tumor growth, and metastases, and shorten survival in animal models [25]. Moreover, people infected with HIV have a substantially higher risk on some types of cancer compared to uninfected people of the same age [26]. In the case of NGF signaling pathway, it has been shown to alter cell death and survival in various cancer cells [27]. Finally, several different review articles have been published on the role of EGFR in the pathogenesis of human carcinoma and it was proposed as a potential novel therapeutic target [28].

In the case of breast cancer, we have found three exclusive pathways, including Cdc20: phospho-APC/C mediated degradation of cyclin A, regulation of beta-cell development and biological oxidations. Cdc20 may function as an oncoprotein. Several studies have shown that Cdc20 is highly expressed in various types of human tumors. It has been reported that Cdc20 is over-expressed in breast cancer cells compared to normal mammary epithelial cells [29]. Finally, the progression of breast cancer has been associated with the oxidative stress from several studies [30].

For the cases of colorectal, glioma and ovarian cancer, there have been found exclusive pathways including the membrane trafficking pathway for the colorectal, inhibition of HSL for the glioma and wnt signaling pathway for the ovarian cancer, respectively. Membrane trafficking proteins constitute novel targets in the treatment of metastatic colorectal cancer [31]. Wnt plays an important role in ovarian cancer and it has been proposed as a potential target in the development of new drugs for ovarian cancer as a single agent and in combination with chemotherapy or other targeted agents [32].

### Informed walks and drug repurposing

The highlighted sub-networks produced with the Informed Walks method were further processed in order to investigate their contribution to the discovery of potential drugs for the different cancer types. Actually, the genes that constitute the sub-networks from each cancer type were divided into up and down regulated, based on their fold change from the initial statistical analysis of the TCGA datasets. The up and down regulated genes formed disease signatures that were queried in the well-established drug repurposing pipeline LINCS-L1000, The Library of Network-Based Cellular Signatures (http://www.lincscloud.org/), currently replaced by Clue (https://clue.io/). This pipeline is the advanced version of Connectivity Map (cMap) [33] with significantly increased number of drug treatments, cell types and gene signatures based on L1000 high throughput technology. We used the LINCS-L1000 detailed report and we collected the top 20 drugs for each cancer type with the most negative enrichment scores. The negative score suggests that the drugs are considered to be inhibitors. Common and exclusive drugs of each cancer type were further investigated (Additional file 1: Table S18).

**Fig. 3:**
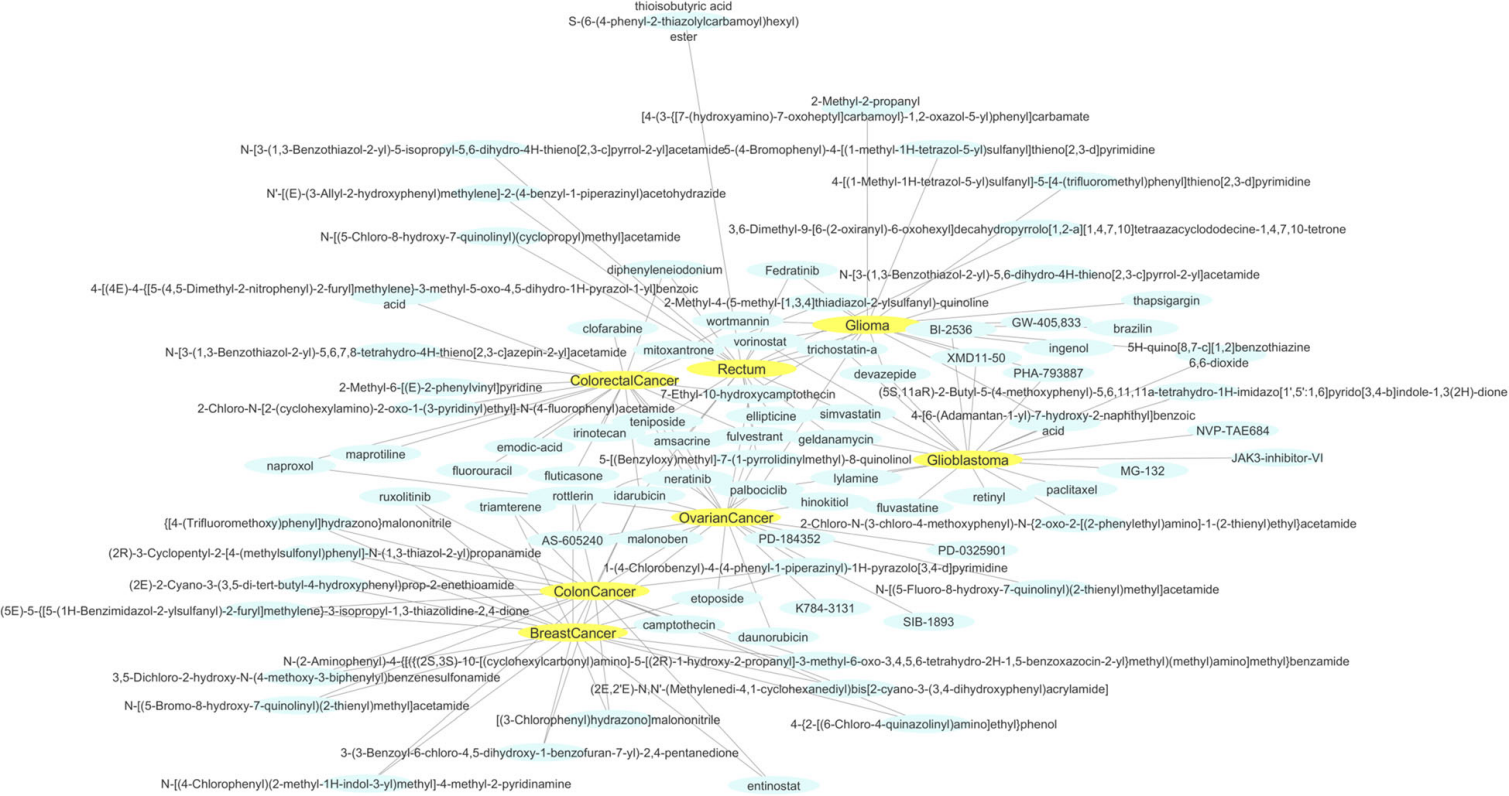
Drug – Disease Network. Nodes with blue color represent the repurposed drugs of each cancer type and nodes with yellow color represent the seven different cancer types

One repurposed drug - Idarubicin was found as common (Fig. 3) in four out of seven cancer types (breast cancer, colorectal cancer, ovarian cancer and rectum). Idarubicin is a chemotherapy drug used to treat some types of cancer including acute myeloid leukemia and advanced breast cancer. Furthermore, two repurposed drugs, fulvestrant and amsacrine, were found as common in three cancer types of the same family (colon cancer, colorectal cancer and rectum) as well as in ovarian cancer.

It has been reported that basal-like or triple negative breast cancer subtype and serous ovarian cancer have important genomic similarities. The mutation spectrum (the types and frequencies of genomic mutations) was largely the same in both cancer types. Further analyses identified several additional common genomic features, such as gene mutation frequency, suggesting that diverse genomic aberrations can converge into a limited number of cancer subtypes (http://www.cancer.gov). Pyrazoles, an organic compound, was found as a common repurposed drug against both ovarian and breast cancer. Furthermore, two repurposed drugs (trichostatin-a and vorinostat) were found as common in four out of seven cancer types (colon cancer, glioblastoma, glioma and rectum). It has been found that the MCM-2 target gene of trichostatin-a is a novel therapeutic target in colon cancer cells [34]. Moreover, three cancer types from the same family (colon cancer, colorectal cancer and rectum) share a common repurposed drug – diphenyleneiodonium. It has been reported that diphenyleneiodonium have therapeutic potential for NADPH oxidase-containing human colon cancers in vivo and that at least part of their antineoplastic mechanism of action may be related to targeting Nox1 [35].

As shown in Fig. 3, six common repurposed drugs were found as common for glioma and glioblastoma (devazepide, PHA-793887, 4-[6-(Adamantan-1-yl)-7-hydroxy-2-naphthyl]benzoic acid, 5H–quino[8,7-c] [1, 2]benzothiazine 6,6-dioxide, XMD11–50, BI-2536). Among these common repurposed drugs, BI-2536 is a PLK1 inhibitor and it has been reported that PLK1 level is elevated in glioblastoma multiforme (GBM) and its inhibition restricts the growth of brain cancer cells [36].

In the case of exclusive repurposed drugs, 17 were found as exclusive for breast cancer, 9 for colon cancer, 8 for colorectal cancer, 6 for rectum, 10 for ovarian cancer, 9 for glioblastoma and 7 for glioma. From these drugs, hinokitiol and etoposide were found as exclusive for ovarian cancer. Hinokitiol is a natural compound which may act as an anti- asculogenic mimicry agent, and it has been reported that may be useful for the development of novel breast cancer therapeutic agents [37]. Moreover, treatment with etoposide is generally effective and well-tolerated in platinum-resistant ovarian cancer patients [38].

Furthermore, ruxolitinib, a drug for the treatment of intermediate or high-risk myelofibrosis, was found as an exclusive repurposed drug for breast cancer. An ongoing clinical trial (October, 2015) has compared the overall survival of women with advanced (Stage III) or metastatic (Stage IV) HER2-negative breast cancer who received treatment with capecitabine in combination with ruxolitinib versus those who received treatment solely with capecitabine (https://clinicaltrials.gov) [4]. Moreover, an exclusive breast cancer repurposed drug, rottlerin, was found that leads to the apoptosis in breast cancer stem cells [39].

In the case of colon cancer, Amino-purvalanol A was found as an exclusive repurposed drug. Amino-purvalanol A is a cell-permeable cyclin-dependent kinase inhibitor that arrests cell cycle at G2/M boundary (IC50 = 1.25 μM) and induces apoptosis at concentrations greater than 10 μM. A very similar compound, purvalanol A, potently suppresses the anchorage-independent growth of c-Src-transformed cells as well as HT-29 and SW48 human colon cancer cells [40]. Moreover, irinotecan, an exclusive repurposed drug that was found in the case of colorectal cancer, in combination with fluorouracil and leucovorin benefits patients with metastatic colorectal cancer [41]. From the rectum analysis, mitoxantrone is one out of six exclusive drugs that were found. Mitoxantrone is used to treat certain types of cancer, mostly metastatic breast cancer, acute myeloid leukemia, and non-Hodgkin’s lymphoma.

In the case of glioblastoma, MG-132 is found as an exclusive repurposed drug. This is a proteasome inhibitor, it has been reported that induces selective apoptosis in glioblastoma cells through inhibition of PI3K/Akt and NFkappaB pathways, mitochondrial dysfunction and activation of p38-JNK1/2 signaling [42]. Finally, brazilin, a repurposed drug that was found as exclusive for glioma, it has been reported that inhibits growth and induces apoptosis in Human glioblastoma Cells [43].

### Informed walks and functional performance

We tried to compare our method with other methods based on the network topology and more specifically on the centrality measures. More specifically, the top 1000 genes (using LIMMA R package [44] with *p*-value <0.05 and sorted based on the absolute value of their log Fold Change) were used in order to construct a co-expression network for each cancer type. Using the igraph R package, we calculated the degree, the betweenness and the closeness centrality of the top 1000 genes for each cancer type. In order to have the same number of genes to compare with the Informed Walks, the top 574 genes for breast cancer, top 547 for colon cancer, top 593 for colorectal cancer, top 523 for rectum, top 585 for ovarian cancer top 544 for glioblastoma and top 536 genes for glioma based on the three centrality measures were used for further analysis.

For each cancer type, the four different ranked lists, namely the lists from the Informed Walks, the degree centrality-based ranking, the betweenness centrality-based ranking and the closeness centrality-based ranking, were used as input in the ToppGene Suite (https://toppgene.cchmc.org/prioritization.jsp) [45], a one-stop portal for gene list enrichment analysis and candidate gene prioritization based on functional annotations and protein interactions network. ToppGene, works by taking as input a training and a test set of genes and it computes a similarity score between the two sets based on semantic annotations. In our analysis we used as training set the most related genes to each cancer type according to the Malacards human disease database (http://www.malacards.org) [46] and as test set each ranked gene list from the Informed Walks method and the network centralities. We used 3 categorical terms: pathway, interactions and co-expression to prioritize the genes for each test set. Table 1 presents the average similarity score for the 3 categorical terms for each method and cancer type and the number of significant genes (*p*-value < 0.05) of the test set, derived by random sampling from the whole genome. It is notable that both the maximum average functional similarity score and the maximum number of significant genes, for all cancer types, were achieved by Informed Walks.

**Table 1:** Functional scores of gene lists derived from Informed Walks and network centrality measures

	Prioritization based on Informed Walks		Prioritization based on Degree centrality		Prioritization based on Betweenness centrality		Prioritization based on Closeness centrality	
	Average of Functional Similarity Score	Number of significant genes	Average of Functional Similarity Score	Number of significant genes	Average of Functional Similarity Score	Number of significant genes	Average of Functional Similarity Score	Number of significant genes
*Breast*	0.968	186	0.949	92	0.947	97	0.941	100
*Colon*	0.771	179	0.682	84	0.683	76	0.673	80
*Colorectal*	0.983	153	0.939	63	0.948	56	0.957	56
*Rectum*	0.566	41	0.381	8	0.402	12	0.392	15
*Ovarian*	0.914	252	0.873	131	0.873	112	0.866	116
*Glioma*	0.875	284	0.861	75	0.829	102	0.823	106
*Glioblastoma*	0.905	287	0.873	118	0.870	113	0.869	100

## Discussion and conclusions

In this work we presented the Informed Walks method and applied the corresponding model to gene co-expression networks of seven different cancer types in order to examine the contribution of this approach to the identification of significant genes and gene-gene links related to each cancer type. We further analyzed each derived sub-network, in order to investigate potential mechanisms and drugs specifically for breast cancer, colon cancer, colorectal cancer, rectum, ovarian cancer, glioblastoma and glioma. As described in the previous section, we have found 10 common pathways for the seven different cancer types including axon guidance, cell cycle checkpoints, signaling by FGFR, DNA repair, DNA replication, opioid signaling, HIV infection, cell cycle, mitotic signaling by NGF and signaling by EGFR. These mechanisms have a pivotal role in cancer development and tumorigenesis and may be potential therapeutic targets (signaling by EGFR). We have also found exclusive pathways for each cancer type. In the case of breast cancer, we have found three exclusive pathways, including Cdc20: Phospho-APC/C mediated degradation of cyclin A, regulation of beta-cell development and biological oxidations. It has been reported that Cdc20 and securin are promising candidates for clinical applications in breast cancer prognostication, especially in the challenging prognostic decisions of triple negative breast cancer. Membrane trafficking has been found as an exclusive pathway for colorectal cancer. It has been reported that membrane receptors constitute novel targets during current treatment of metastatic colorectal cancer (CRC) due to the fact that their aberrant expression/activity favors cancer cell properties [31]. Inhibition of HSL and Wnt signaling pathways, have been found as exclusive for glioma and ovarian cancer respectively. It is widely known that the Wnt/β-catenin signaling pathway has been considered to be a factor in the development and progression of ovarian cancer [47].

The derived sub-networks were also analyzed by means of LINCS drug repositioning pipeline, proposing potential anticancer drugs for each cancer type. Based on the analysis, we concluded to 17 exclusive out of 20 repurposed drugs for breast cancer, 9 for colon cancer, 8 for colorectal cancer, 6 for rectum, 10 for ovarian cancer, 9 for glioblastoma and 7 for glioma. As described above in the case of breast cancer, one exclusive drug (ruxolitinib), is on an ongoing clinical trial in combination with capecitabine for the survival of women with advanced (Stage III) or metastatic (Stage IV) HER2-negative breast. Moreover, entinostat in combination with nivolumab and ipilimumab is on a clinical trial (recruiting participants) for the examination of their ability in treating patients with solid tumors that are metastatic or cannot be removed by surgery or locally advanced or metastatic HER2-negative breast cancer (ClinicalTrials.gov). In the case of colon cancer, the exclusive repurposed drug selumetinib has been examined in a clinical trial in Phase I in combination with MEDI4736 in order to investigate the safety, tolerability, pharmacokinetics and anti-tumour activity of ascending doses in patients with advanced solid tumors(ClinicalTrials.gov). Irinotecan has been found as an exclusive drug for colorectal cancer. An ongoing clinical trial (Phase 1) has evaluated the low-dose irinotecan and stereotactic body radiotherapy to treat colorectal cancer with limited liver metastasis. Finally, fluvastatin (found to be an exclusive drug for glioblastoma), is examined for its safety in low and high grade optico-chiasmatic gliomas (ClinicalTrials.gov).

Despite using the whole spectrum of genes (17,814) instead of a subset with disease specific genes (i.e. top 1000), Informed Walks model managed to highlight cancer specific sub-networks, as well as mechanisms and genes that are already associated with cancer types in the literature. Furthermore, its contribution in the investigation of repurposed drugs was quite high, as several drugs are already in ongoing clinical trials.

Finally, the dominance of Informed Walks is profound in the analysis of the functional performance of the derived gene lists for each cancer type, compared to the functional performance of the gene lists derived from network centrality analysis.

The action of the remaining mechanisms and drugs proposed by the LINCS may be further investigated, since they have been derived from genes significantly related to each cancer type.

We would like to highlight that, in a future direction, our method could provide even deeper information if it were applied to single cell analysis data. Actually, at this stage, the analysis is performed on a group of various cell types in each tissue sample under the assumption that all cells of a particular type are identical. It is worth to note that individual cells within the same population may significantly differ (a percentage of them could be non-cancerous) and these differences can have an impact to the resulted mechanisms.

## Methods

### Datasets and preprocessing

TCGA mRNA (microarray) gene expression data for seven cancer types have been obtained from Firehose (http://gdac.broadinstitute.org/). More specifically, we collected all the available datasets with both normal and tumor mRNA gene expression data (Table 2). At the period (March 2016) of data retrieval from the above website, only seven cancer types were containing mRNA gene expression data from tumor and normal patients. Each dataset contains expression data of 17,814 genes. The seven distinct TCGA datasets were statistically analyzed with the LIMMA (Linear Models for Microarray Data) R package in order to find the Differentially Expressed Genes (DEGs) in tumor samples compared to the normal ones [44].

**Table 2:** TCGA datasets with normal and tumor samples

*Cancer Types*	Total Samples	Normal Samples	Disease Samples
*Breast Invasive carcinoma*	587	61	526
*Colon Adenocarcinoma*	172	19	153
*Colorectal Adenocarcinoma*	244	22	222
*Glioblastoma multiforme*	512	10	502
*Glioma*	539	10	529
*Ovarian serous cystadenocarcinoma*	580	8	572
*Rectum adenocarcinoma*	72	3	69

### Network reconstruction

A network inference method, based on mutual information, was used in order to reconstruct a co-expression network for each cancer type using 17,814 genes by means of MRNETB (Maximum Relevance Minimum Redundancy Backward) algorithm- an improved version of the network inference algorithm MRNET (Maximum Relevance Minimum Redundancy) [48]. MRNET applies a forward selection strategy to identify a set of neighbors for every variable. However, forward selection methods suffer in performance if the first neighbor is chosen incorrectly. On the other hand, MRNETB implements a combination of backward elimination and a sequential replacement procedure keeping the same computational cost [48]. The selected method is implemented in R package. Specifically, we used the PARMIGENE (PARallel Mutual Information calculation for GEne NEtwork reconstruction) R-package which provides a parallel estimation of the mutual information based on entropy estimates from k-nearest neighbors distances [49], in order to calculate the mutual information and the MINET (Mutual Information NETworks) R-package [50] for the MRNETB algorithm.

Subsequently, we applied an edge filtering scheme to each of the reconstructed networks in order to remove the low weighted gene - gene links. More specifically, we iteratively filtered the networks, by removing a percentage of the weakest edges compared to the maximum weight for each network, until the maximum fully connected sub-network was generated. Finally, we concluded to 7 sub-networks containing the whole set of genes (17,814 genes-nodes) and a number of edges of the order of ~ 10 million.

### Informed walks

**Fig. 4:**
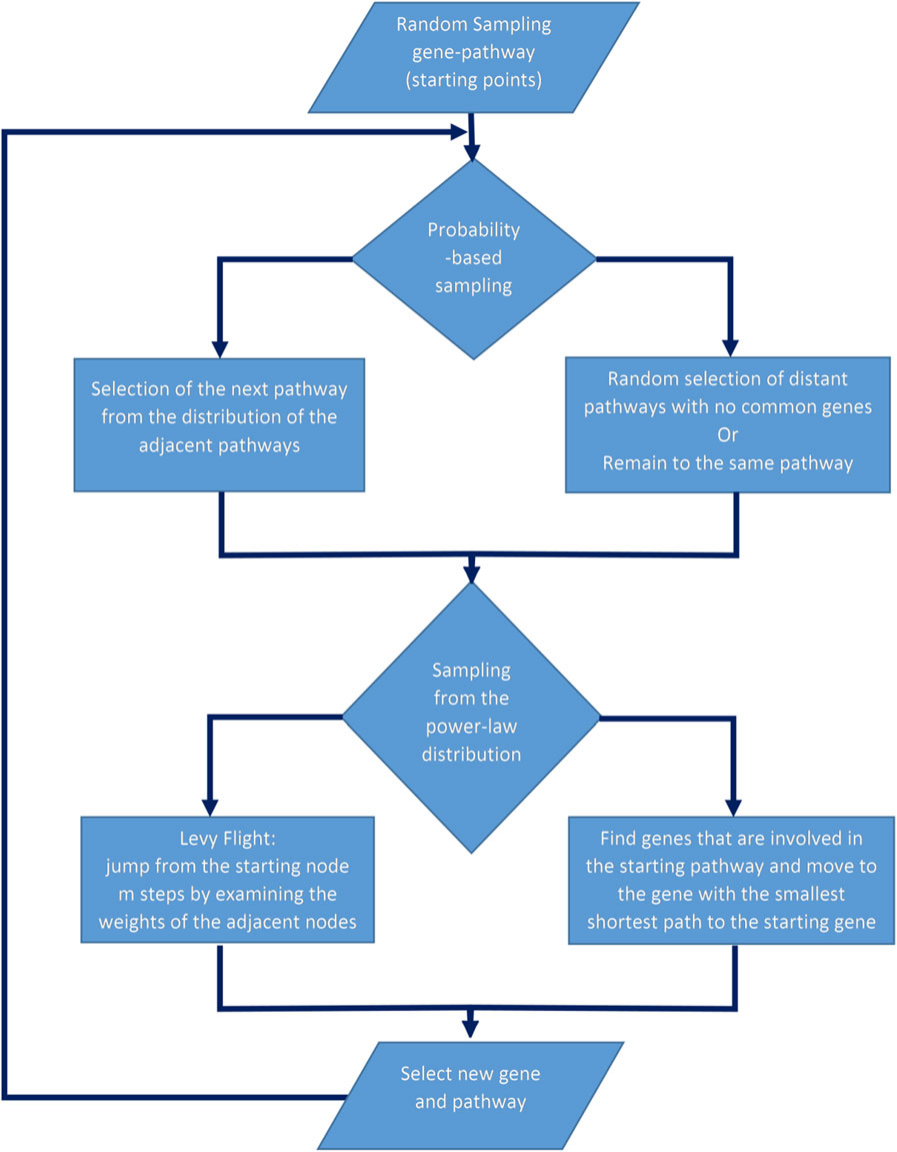
Flowchart presenting the Informed Walks procedure

When randomly walking on a network, even with restarts, there are some issues that may trap the agent (random walker) or make the agent’s reporting unimportant. Specifically, if the agent’s walk is defined only by the network characteristics then the agent’s reporting will be, at the very best, a good approximation of the network topology that is already known. However, it will be impossible for the agent to provide any evidence regarding the hidden functionality within the walked subnetworks. Further to this, the agent may be entrapped to walk in dense neighborhoods in the network without being capable to select a way-out to other, more isolated, neighborhoods. Trying to overcome these limitations, we used random walks with restarts incorporating information from a molecular pathway network. The latter was generated by connecting pathways that share common genes incorporating the Reactome pathways with their corresponding genes (Fig. 4) from the ConsensusPathDB database (http://consensuspathdb.org/) [51]. The walkers scuff in the co-expression network using a map that contains information regarding pathway associations. More specifically, the walkers switch from one node to another by examining if two nodes/genes are involved in the same pathway. Then they use the shortest path in order to switch from a node/gene to another. To avoid the entrapments in certain neighborhoods we used the concept of Levy flights [52]. Following this approach, the walker investigates the nearby area by sampling from a power law distribution and, depending on the derived sample, the walker jumps or not to another area in the network by a number of steps defined from the sampling.

Deepening in detail, initially the algorithm selects a random gene from the co-expression network and a pathway from the pathway network (starting points). Subsequently, the algorithm identifies all the genes that are involved in the specific pathway. The shortest paths from the initial gene to each gene of the same pathway are calculated and the walker moves to the gene with the minimum shortest path. The next pathway is selected through a Monte Carlo decision that favors (with a 70% probability) the pathways that contain more common genes with the current pathway. However, there is a 30% probability to choose a pathway with no common genes with the current one or even choose again the same pathway with random sampling.

**Fig. 5:**
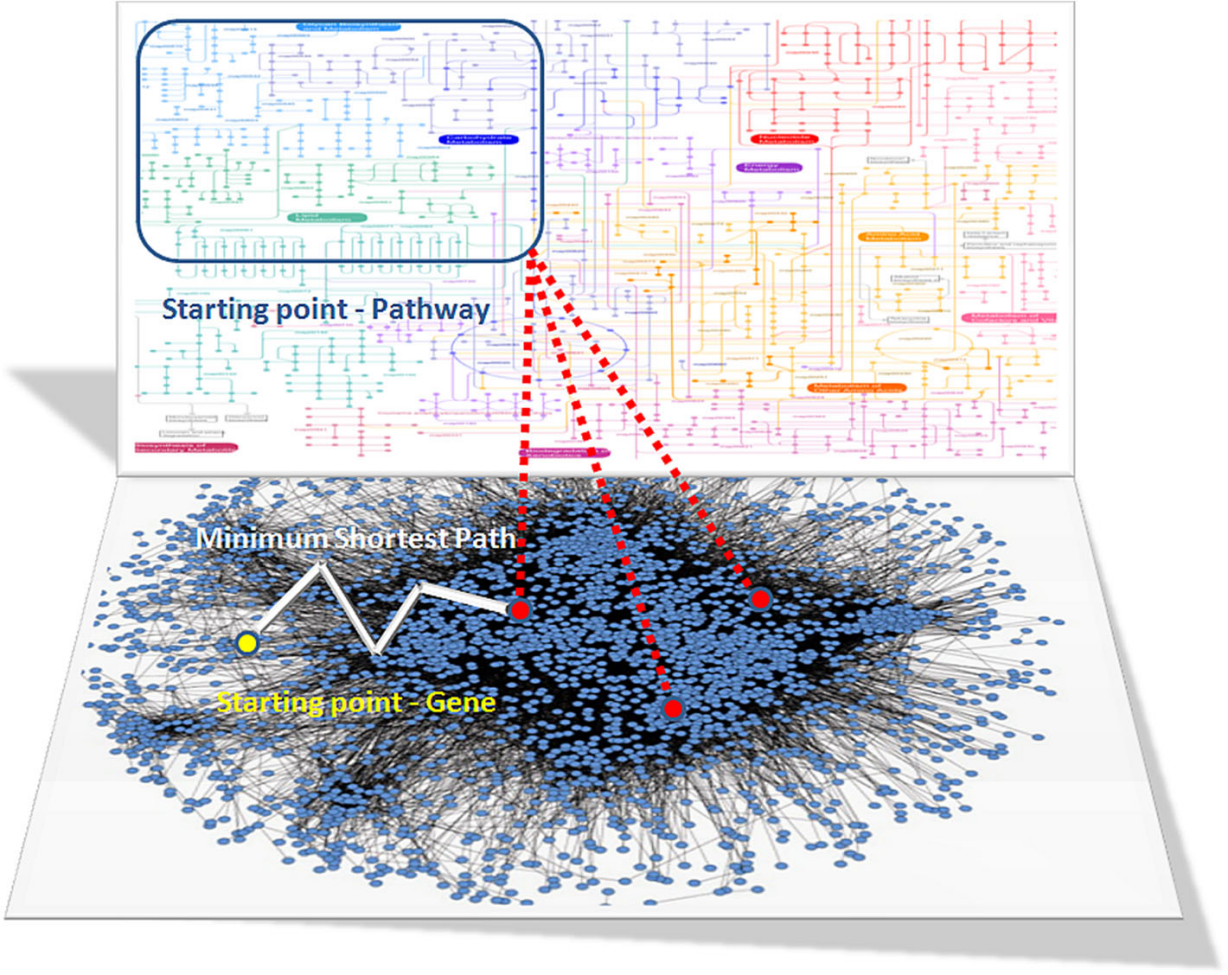
The layout of the Informed Walks model. Starting from a randomly selected gene and pathway (Starting Points), the algorithm identifies all genes that are involved in the specific pathway. The shortest paths from the starting point/gene (yellow color) to each gene of the pathway (red color) are calculated and the walker moves to the gene with the minimum shortest path

Usually, the walker searches for genes that are involved in the starting pathway and moves to the gene/node with the smallest shortest path from the current gene/node. However, driven by a power law distribution sampling, the walker is able to perform Levy flight by moving to another area of the co-expression network by a sampled number of steps away from the starting gene, preferably passing from the edges with the highest weight each time. The total number of genes (N) in the network defines the various thresholds and constraints of this Levy flight. Finally, by integrating each walker’s passing edges, a new network is derived having the same nodes/genes as the initial gene co-expression network. Nevertheless, the edges of this network represent the total passage frequency of all the walkers between each pair of nodes. From this network, we derived a subnetwork with the top 500 edges for each cancer type. The flowchart of the methodology and the visualization of the Informed Walks procedure are presented in Figs. 4 and 5 respectively.

The Informed Walks model has been implemented in R programming language. The input files were the co-expression network for each cancer type and the pathway network. More specifically, the Informed Walks model was applied to the large-scale gene co-expression network that contained 17,814 node/genes and ~ 10 million edges. For each cancer type, the Informed Walks algorithm was executed for 1000 iterations with 300 restarts (walkers). The demanding computations were performed on ‘ARIS’ National High Performance Computing Infrastructure of the Greek Research and Technology Network. Specifically, each walker was allocated to run in parallel in 20 separate nodes, each one processing 15 different tasks and requiring 48GB of RAM.

## Additional file

Additional file 1: Table S1. Common and Exclusive genes between the seven subnetworks for each different cancer type. **Table S2.** Significant pathways for the case of common genes between the seven cancer types. **Table S3.** Significant pathways for the case of the exclusive breast cancer genes. **Table S4.** Significant pathways for the case of the exclusive colon cancer genes. **Table S5.** Significant pathways for the case of the exclusive colorectal cancer genes. **Table S6.** Significant pathways for the case of the exclusive rectum genes. **Table S7.** Significant pathways for the case of the exclusive ovarian cancer genes. **Table S8.** Significant pathways for the case of the exclusive glioma genes. **Table S9.** Significant pathways for the case of the exclusive glioblastoma genes. **Table S10.** Significant pathways for the case of breast cancer. **Table S11.** Significant pathways for the case of colon cancer. **Table S12.** Significant pathways for the case of colorectal cancer. **Table S13.** Significant pathways for the case of rectum. **Table S14.** Significant pathways for the case of ovarian cancer. **Table S15.** Significant pathways for the case of glioblastoma. **Table S16.** Significant pathways for the case of glioma. **Table S17.** Common and Exclusive mechanisms between the seven different cancer types. **Table S18.** Common and exclusive repurposed drugs of each cancer type. (DOCX 52 kb)

## Abbreviations

*cMap*: Connectivity Map
*DEGs*: Differentially Expressed Genes
*LIMMA*: Linear Models for Microarray Data
*LINCS*: The Library of Network- Based Cellular Signatures
*MINET*: Mutual Information NETworks
*MRNET*: Maximum Relevance Minimum Redundancy
*MRNETB*: Maximum Relevance Minimum Redundancy Backward
*Parmigene*: PARallel Mutual Information calculation for GEne NEtwork reconstruction
*TCGA*: The Cancer Genome Atlas
