# Supplementary material for: Informed walks: whispering hints to gene hunters inside networks’ jungle
Source: BMC Syst Biol. 2017 Oct 11;11:97. doi: 10.1186/s12918-017-0473-6 (PMC5637247; doi:10.1186/s12918-017-0473-6)
Supplement: Additional file 1: Table S1. — Common and Exclusive genes between the seven subnetworks for each different cancer type. Table S2. Significant pathways for the case of common genes between the seven cancer types. Table S3. Significant pathways for the case of the exclusive breast cancer genes. Table S4. Significant pathways for the case of the exclusive colon cancer genes. Table S5. Significant pathways for the case of the exclusive colorectal cancer genes. Table S6. Significant pathways for the case of the exclusive rectum genes. Table S7. Significant pathways for the case of the exclusive ovarian cancer genes. Table S8. Significant pathways for the case of the exclusive glioma genes. Table S9. Significant pathways for the case of the exclusive glioblastoma genes. Table S10. Significant pathways for the case of breast cancer. Table S11. Significant pathways for the case of colon cancer. Table S12. Significant pathways for the case of colorectal cancer. Table S13. Significant pathways for the case of rectum. Table S14. Significant pathways for the case of ovarian cancer. Table S15. Significant pathways for the case of glioblastoma. Table S16. Significant pathways for the case of glioma. Table S17. Common and Exclusive mechanisms between the seven different cancer types. Table S18. Common and exclusive repurposed drugs of each cancer type. (DOCX 52 kb) [file 12918_2017_473_MOESM1_ESM.docx]

**Supplementary Materials**

**Informed walks: whispering hints to gene hunters inside networks’ jungle**

Marilena M. Bourdakou^1,2**^ and George M. Spyrou^1*^

^1^ Bioinformatics ERA Chair, The Cyprus Institute of Neurology and Genetics, 6 International Airport Avenue, Ayios Dometios, 2370 Nicosia, Cyprus

^2^ Center of Systems Biology, Biomedical Research Foundation, Academy of Athens, Soranou Ephessiou 4, 115 27 Athens, Greece.

^*^ Corresponding author. George M. Spyrou, Tel.: (+357) 22 392 852; E-mail: [georges@cing.ac.cy](mailto:georges@cing.ac.cy)

^**^ Marilena M. Bourdakou, E-mail: [mbourdakoy@bioacademy.gr](mailto:mbourdakoy@bioacademy.gr)

**Supplementary Table 1:** Common and Exclusive genes between the seven subnetworks for each different cancer type

| **Cancer Types** | **Common Genes** | **Genes** |
| --- | --- | --- |
| Breast Colon Colorectal Glioblastoma Glioma Ovarian Rectum | 70 | BID,HIST1H4J,CCNB1,UBB,CD4,HIST1H4E,GJA1,PLK1,MAOA,GJB1,CDKN1B,RASAL1,CD36,RPS27A,GNG12,NUP88,PPP2R1B,NRAS,POLA1,NGFR,EGF,PCNA,HIST1H4L,MYH11,SPRED1,AGER,HSPA1A,PPIA,PRKAG3,HRAS,ADCY8,TGFB1,RFC4,CALM2,HIST1H4K,CASP3,AAAS,HIST1H4B,WNT1,PRKAR2B,MOV10,STX1A,PLCB1,AKT2,APOBEC1,FGF9,HIST1H4D,PPP2R1A,RPA1,DYNLL1,NCBP2,UBA52,SPTB,NCBP1,HIST1H4F,CALM3,TP53,CDKN1A,HIST1H4H,AKT1,KLC2,SPRED2,HIST1H4A,HIST1H4C,CALM1,RFC5,PAK2,FGF23,ERCC2,UBC |
| Breast Colon Colorectal Glioblastoma Ovarian Rectum | 1 | SYT1 |
| Breast Colon Colorectal Glioma Ovarian Rectum | 1 | FGF2 |
| Breast Colon Colorectal Glioblastoma Glioma Rectum | 2 | GNG10,UBE2D1 |
| Breast Colon Colorectal Glioblastoma Glioma Ovarian | 9 | SOS1,NTRK1,TNFSF10,FGF8,AGT,PTGS2,TGFBR1,PSIP1,TNRC6B |
| Breast Colon Glioblastoma Glioma Ovarian Rectum | 4 | TRAF6,EGFR,RASAL2,COMT |
| Breast Colorectal Glioblastoma Glioma Ovarian Rectum | 3 | PRKAR2A,ATM,HDAC1 |
| Colon Colorectal Glioblastoma Glioma Ovarian Rectum | 7 | RAC1,RFC3,PLCG1,FRS2,HIST1H4I,RPA3,POLA2 |
| Breast Colon Colorectal Ovarian Rectum | 12 | OR4A15,FGF20,MAD2L1,FYN,CASP1,GLYATL1,FGF18,INHBC,S100A12,MPG,AKT3,TRIM62 |
| Breast Colon Colorectal Glioblastoma Ovarian | 2 | CYP2J2,PYCARD |
| Breast Colon Colorectal Glioma Ovarian | 1 | CDK2 |
| Breast Colon Colorectal Glioblastoma Glioma | 3 | CREB1,TGFBR2,NUP62 |
| Breast Colon Glioblastoma Glioma Ovarian | 2 | FGFR3,CHRNA3 |
| Breast Colorectal Glioblastoma Ovarian Rectum | 2 | TOR2A,SRC |
| Breast Colorectal Glioblastoma Glioma Rectum | 1 | GHSR |
| Breast Colorectal Glioblastoma Glioma Ovarian | 2 | SPINK7,DUSP6 |
| Breast Glioblastoma Glioma Ovarian Rectum | 12 | RPS11,RPA2,SNAP25,ITGB1,CALR,CD300LG,PIK3CD,APEX1,IKBKB,GTF2F1,SV2A,CYP8B1 |
| Colon Colorectal Glioblastoma Ovarian Rectum | 1 | ST6GAL1 |
| Colon Colorectal Glioma Ovarian Rectum | 1 | PRKAG1 |
| Colon Colorectal Glioblastoma Glioma Rectum | 10 | BRCA1,ADRA2A,C10orf76,SLBP,MYH10,ERCC3,ALOXE3,GJB2,CBLN3,PTCH2 |
| Colon Colorectal Glioblastoma Glioma Ovarian | 4 | KLK14,MAP3K8,PROS1,IZUMO1 |
| Colon Glioblastoma Glioma Ovarian Rectum | 2 | MUTYH,ABL1 |
| Colorectal Glioblastoma Glioma Ovarian Rectum | 1 | RPL22 |
| Breast Colon Colorectal Rectum | 4 | APOB,RFX1,MYADML,XPO1 |
| Breast Colon Colorectal Ovarian | 8 | CGA,CRP,HUWE1,DHX9,ACSM1,TAAR5,MCF2,APH1B |
| Breast Colon Colorectal Glioblastoma | 1 | F7 |
| Breast Colon Colorectal Glioma | 1 | RHAG |
| Breast Colon Ovarian Rectum | 4 | SUMO1,OR2H2,PLA2G4D,FKSG83 |
| Breast Colon Glioma Rectum | 1 | NOS3 |
| Breast Colon Glioblastoma Ovarian | 1 | FDXR |
| Breast Colon Glioblastoma Glioma | 7 | CHUK,HSP90AA1,PPP3R1,IGKC,PI4K2B,BUB1B,FGF21 |
| Breast Colorectal Ovarian Rectum | 2 | MED25,ADCY4 |
| Breast Colorectal Glioblastoma Ovarian | 2 | CCNA1,MAOB |
| Breast Colorectal Glioma Ovarian | 3 | IL12RB1,ECHS1,AQP7 |
| Breast Colorectal Glioblastoma Glioma | 2 | NUP133,KCNJ12 |
| Breast Glioblastoma Glioma Rectum | 4 | TCL6,ADD1,GRIN1,CCNT1 |
| Breast Glioblastoma Glioma Ovarian | 7 | PIK3CB,GLYATL2,ADAM10,FOXO1,PIK3CA,KLB,HMGB1 |
| Colon Colorectal Ovarian Rectum | 8 | FBXO11,HIST1H3B,FGF19,CCNA2,PAOX,HNRPK,MYOG,UGP2 |
| Colon Colorectal Glioblastoma Rectum | 1 | HSPA8 |
| Colon Colorectal Glioma Rectum | 3 | SHC1,FGF22,SCN2B |
| Colon Colorectal Glioblastoma Glioma | 8 | RAP1A,COPA,IRF3,TNKS,CD9,MAPK8,TNRC6C,SPRYD3 |
| Colon Glioblastoma Glioma Rectum | 1 | OCRL |
| Colon Glioblastoma Glioma Ovarian | 3 | SEMA3A,FGF4,BANF1 |
| Colorectal Glioblastoma Ovarian Rectum | 1 | PLA2G4C |
| Colorectal Glioma Ovarian Rectum | 2 | OR8U1,TCEB2 |
| Colorectal Glioblastoma Glioma Rectum | 2 | MAPK1,ARAF |
| Colorectal Glioblastoma Glioma Ovarian | 9 | VAMP2,ALDH2,CDH15,PLA2G4A,NUP85,STAT1,ADAR,GNG11,NOTCH1 |
| Glioblastoma Glioma Ovarian Rectum | 8 | CYCS,CDC6,HCK,ACTN2,TFAM,ADAM17,PTGS1,HSPG2 |
| Breast Colon Colorectal | 16 | C11orf42,AQP2,CENPO,HSD3B1,MOBKL2A,OR10H2,GRIA3,DUSP11,GAPVD1,LALBA,PUM2,WIPF2,DIABLO,RFC1,FGF5,KHDRBS1 |
| Breast Colon Rectum | 4 | MAP3K7IP1,MLLT1,UBE2O,IFIH1 |
| Breast Colon Ovarian | 4 | PLK4,FAM134C,HDAC2,RNF151 |
| Breast Colon Glioblastoma | 1 | ILF3 |
| Breast Colorectal Rectum | 1 | RHOG |
| Breast Colorectal Ovarian | 8 | MED12,KCTD5,LOC643905,CDK5R2,SFRS2,KLHL18,IL10RA,HSD17B12 |
| Breast Ovarian Rectum | 12 | CLRN1,GPR3,OR7D4,OR52D1,PCK1,LCE2B,SMR3A,CHD2,RAPGEF2,NFKBIA,CYP46A1,GRIA2 |
| Breast Glioblastoma Rectum | 2 | CCDC114,MYH14 |
| Breast Glioblastoma Ovarian | 7 | DNM1,ACPT,STAT3,CTH,FGFR4,GRIK1,ITGB3 |
| Breast Glioma Ovarian | 4 | ESPL1,PGLYRP1,RASGRP1,HSPA5 |
| Breast Glioblastoma Glioma | 16 | PTPN11,OR4D10,ANAPC10,ASB15,LMNB1,ACP5,TNRC6A,SLC22A7,UCP1,AVPR1A,OTUD5,CDC16,ADCY2,MLXIPL,GNG13,ALB |
| Colon Colorectal Rectum | 22 | VPS4A,TMEM127,YME1L1,RAD9A,CYP2A6,UMPS,PTPN23,HNRPUL1,CRHR1,TDG,MARK2,PRDM10,SLC9A1,SAA1,HIF1AN,MGC21874,SLC25A45,AFTPH,WWP2,SAA2,DDX20,ELAVL3 |
| Colon Colorectal Ovarian | 6 | RIC8A,CDCA5,ATF2,C7orf43,C5orf27,ZBTB40 |
| Colon Colorectal Glioblastoma | 3 | FGFR1,KRTAP11-1,SF3A1 |
| Colon Colorectal Glioma | 2 | ACSL1,IL1B |
| Colon Ovarian Rectum | 1 | UBTF |
| Colon Glioblastoma Ovarian | 2 | SLC7A8,CFB |
| Colon Glioblastoma Glioma | 12 | PANX1,PLCB2,NOSIP,SCP2,TSN,GPR132,HDAC3,OR10R2,IL6,CUL1,DIO2,CHERP |
| Colorectal Ovarian Rectum | 7 | INSRR,C1orf77,CASP7,APH1A,FAIM2,SFRS7,EIF2AK1 |
| Colorectal Glioblastoma Rectum | 2 | GNAO1,CAMK4 |
| Colorectal Glioma Rectum | 1 | ERCC1 |
| Colorectal Glioblastoma Ovarian | 2 | INHBB,TREML1 |
| Colorectal Glioma Ovarian | 4 | GRB2,NCF1,PRKAG2,APIP |
| Colorectal Glioblastoma Glioma | 5 | FGF17,FAS,KL,SMARCAL1,LOC441251 |
| Glioblastoma Ovarian Rectum | 1 | AP2M1 |
| Glioblastoma Glioma Rectum | 9 | SLC17A1,C18orf12,MAPK14,B2M,AP2S1,LYK5,CASP9,ITCH,CRKL |
| Glioblastoma Glioma Ovarian | 20 | UBE2G1,SLC6A6,RPS6KA6,DLG4,GJA9,CD226,MAD1L1,GNS,GUCY2D,CENPA,H1F0,UCP2,OR52B2,TNP2,FGFR1OP2,HLA-G,HADHB,NFKBIB,MAP3K7,CFLAR |
| Breast Colon | 17 | NUP93,ALDH4A1,SLC25A44,CYP4F8,FCN2,ADCY3,POLR3G,PSEN2,KEAP1,POP4,CSH1,SPAST,RPS6KA2,CCT7,TUBA1C,CA2,CDKN2A |
| Breast Colorectal | 23 | LUZP1,GPR37L1,C17orf73,GSTA4,SLC6A7,C19orf55,FGB,C3orf17,EIF3H,GFRA3,OR4X2,TUBB6,NTN1,UBE2E1,DBF4B,ABCF3,CD247,FXYD1,SV2C,GSK3A,ATCAY,MCM8,CYP24A1 |
| Breast Rectum | 22 | SOX21,MRPL47,OR1K1,SMOX,CTRB2,RAPSN,TROAP,TULP2,ATP6V1F,INS,TMEM95,FUSIP1,FXYD7,CACNG3,GTDC1,OR5A1,ZFAND3,GNA14,OR10A5,OR1S2,EDG5,ACTR2 |
| Breast Ovarian | 33 | CNGB1,SLIC1,TMEM174,LOC554175,C21orf89,LBP,HIST1H4G,CRHR2,SFRS3,KIF4B,CHRNA2,OPA1,FGFR2,NPAS4,GABRG3,AHCTF1,POLB,DHH,MGC33407,DDN,SLC4A1AP,EBF2,OR5V1,PIK3R1,PABPC1,SLC5A1,EGLN3,ZNF207,KRT85,MMP10,RNF123,HNRNPU,UBR4 |
| Breast Glioblastoma | 2 | TST,ATXN2L |
| Breast Glioma | 7 | ADCY5,EIF5A2,TJP1,HS3ST5,DCP2,NOTCH4,CKAP2L |
| Colon Colorectal | 73 | OTOP2,CSTF1,MON1B,FAM43B,PARN,C9orf165,PCSK1,DIP2A,CLTA,UCRC,FOXE1,CNOT4,YTHDF1,ZNF574,BTRC,DHDDS,TMEM59,FAM135B,SNRPA,RHOA,COP1,PHLPPL,IQSEC2,TDRD10,ZNF384,SH2B1,SPHK1,ATN1,TRA@,ZFYVE20,FBXO5,TMED5,KIR3DL1,DEFA5,ITGB2,NXF1,NHEJ1,MAS1L,ELL,CNOT2,BMPR2,ZFP91,ADAM33,SH3BP2,LSM5,ADORA1,FARSA,GZMB,UBXD2,MKL2,AKR1B1,PPP1CB,C9orf38,RCE1,NOL5A,KIF13B,SUFU,PPP2R2D,RABL3,CDH23,ANKRD53,LAPTM5,ELAVL1,N4BP1,STMN4,POLR3F,HDGF,ALDOB,POLD3,KCNJ16,MTMR3,PGPEP1,PYDC1 |
| Colon Rectum | 12 | AFF4,SET,ATP1B2,SPIN1,PGAP1,C6orf136,PRPF38A,SARDH,DDX23,MED26,MED22,RAB7A |
| Colon Ovarian | 12 | CAND1,LILRB4,TLE2,POL3S,KIAA1183,PRPF31,RPAIN,TARDBP,FCGR1A,HCRT,HIST1H3F,AVP |
| Colon Glioblastoma | 4 | NTRK2,AMMECR1L,RIPK1,FKBP1A |
| Colon Glioma | 1 | PHF20 |
| Colorectal Rectum | 28 | BCL2L12,USF2,CRY2,NOS1,CLDN11,FAM98A,CSF1,DHX33,NCAM1,RAPGEF1,DIXDC1,OR10P1,ASB16,RHCG,C5orf40,2'-PDE,DNAJB13,ZNF317,MICAL2,ZBED4,MTERFD2,GPX5,C3orf35,COX4NB,PSEN1,ANP32B,AAK1,GPR123 |
| Colorectal Ovarian | 17 | SNRP70,NFATC1,RAD23B,PLA2G2A,SMUG1,CASP10,CHMP7,PIK3R3,IL8RA,BLR1,F2,H2AFX,SLC11A1,KLHDC7A,RPS10,ITIH1,C2orf32 |
| Colorectal Glioblastoma | 2 | COX10,NCOR1 |
| Colorectal Glioma | 2 | MOCOS,FCER1G |
| Ovarian Rectum | 17 | MPFL,SMPD2,CTF1,GPATCH8,PHF12,DEFB106B,RHO,OR8G1,CNR2,TMEM173,OR56B1,OR11G2,TRPM3,ERVWE1,LCE2C,TBX6,GNB3 |
| Glioblastoma Rectum | 6 | ASNA1,PLCG2,TMPRSS9,NUPL2,HSD3B2,NOTCH3 |
| Glioma Rectum | 5 | DLG1,ADCY7,C2orf49,ACADS,C10orf26 |
| Glioblastoma Ovarian | 1 | KIR2DS1 |
| Glioma Ovarian | 4 | AQP7P2,OVGP1,SYK,TAAR8 |
| Glioblastoma Glioma | 179 | E2F8,ZAP70,ARRB2,NCF4,LOC643641,LYAR,ALOX5,NRP1,FRY,TRIM22,GPR116,ASCC3,ADAMDEC1,NPHP1,OR51Q1,MKNK1,DVL2,C3,PPM1K,DYNLT1,ARL13B,HLA-DRA,DNMT3B,CCDC99,SYN1,OR2AE1,CNTFR,ARF1,LOC342897,TMEM125,SMC1A,MRE11A,OGG1,OR5AT1,DLG3,RNF20,YWHAZ,FAM22D,SIM1,ZNRD1,HLA-DPA1,NCAPD3,PGM1,SUMO3,HLA-A,HSPA2,AP1M1,SLAMF8,KALRN,TALDO1,C1QTNF7,SULT2A1,PAK3,LRPPRC,ITGA6,GAD2,ZCCHC10,TAC1,ZFPL1,NFATC3,PAPD4,LYZL6,CRAT,GART,NDC80,GIMAP6,PPIAL4,PTGIR,LMX1A,TXLNA,APOBEC3F,FAM91A1,KIR2DL1,KIF5C,CDC45L,KIAA1539,TLR3,CAMK2G,KIAA0556,C3orf27,BMP10,MX2,OR4A16,TIMM8A,WFS1,EXDL1,SYN2,PRPF40A,CCDC102B,EMCN,PAQR7,DNM3,KPNA4,SMCR7L,RPE,SASH1,BRCA2,CLDN5,C3orf63,AGRN,TAF13,AASS,SLC5A5,SPRY1,IPPK,CCNB2,SIRPA,APOH,SMAD1,CEACAM5,MYT1L,CDT1,SLC12A1,CD28,GLUL,FEZ1,ROBO4,HELLS,MAPK3,CCNE2,HMGCL,CDK7,RASGRP3,GAL3ST4,MRPS16,PANX2,RB1,FGD5,ITGAX,RHOJ,PPP2CA,HSPH1,ATP6AP2,SHROOM4,CLSPN,MX1,SLC26A5,HLA-DMA,RPL13A,LOC284352,AP1M2,CCR5,MRCL3,HADHA,XRCC5,RAB1A,CPSF4,ASTN1,MAP2K1,ZNF282,MGC35295,WRNIP1,MTIF2,KCTD21,PLA2G12A,PEX12,NMD3,POLR2E,ALKBH3,TREX1,ACTG1,ODF4,USP44,PEBP1,AOX1,C19orf21,GEMIN5,MBD1,FMN2,GRAMD3,SCO1,RBL1,RBM35B,SLC6A3,OTOP1,IFIT3,SGEF,CDC25A,PLXNA3 |
| Breast | 221 | ANKS1A,KCNK16,PRSS33,OR1L6,PMPCA,FAM65A,HSD17B1,HSPB6,TGFB2,CXCL3,CHRNE,PTEN,TPX2,HMGCS2,SENP1,ATXN7L2,RAB3IP,SLC15A2,GSTCD,SMARCD1,PPM1J,TUSC5,CACNG5,PAPSS2,GLT8D2,CCDC124,EVI5L,KIAA1715,AVPR1B,LMAN1L,SLC15A4,FOXC1,ATAD1,LOC402164,RNF166,DRD3,SON,KIAA0265,NPR1,LETM1,F9,TMEM1,PI4K2A,SGK269,GRIK2,CASP8,IGFBP7,NRP2,EHMT2,KRAS,CHRM1,CHRNB2,ALOX15,IMMT,CEL,PABPC3,MLN,TMEM140,MAP4K1,SLC22A8,BTN2A3,FOXL1,SLC34A3,PLEKHA3,R3HDM2,C3orf22,CENPL,SF3A2,RIF1,SIRPB1,KCNG4,TUBA1A,SNRPC,POLR1A,APAF1,PLAC9,ATP6V0A2,KIF2C,OBFC2B,CIDEC,PSMD12,CEBPZ,BTBD10,NAT13,VDAC3,KCNJ10,GNG2,CDC20,OSTN,SOCS4,RRP1B,STAT6,ITGA2,SHANK3,LRP10,BAT3,BGN,FFAR1,SEH1L,RAPGEF4,C17orf62,HEG1,WHSC2,POLR2D,DTX4,UBASH3A,KIF23,SUCLG1,SLC35A4,CPNE8,ZNF664,WDTC1,CHCHD3,RARA,CENPE,PNPLA3,TTC31,SDPR,ANAPC2,SLC6A1,TUBB4,NPC1L1,C8B,FMNL3,STIL,C12orf43,UBE2C,SLC35D3,LOC440456,SIRT5,RBM42,POLR2F,FOXA1,ACSM2A,ERBB2,OR8B8,CCDC82,AGL,SSB,C1orf166,STAR,TAAR2,hCG_2028557,USP34,STK4,CPNE6,TPO,PMAIP1,GPR113,SYT11,GLYAT,DDC,OR5I1,ALPI,ITM2A,MRGPRE,GPAM,COX7A1,TRIM10,POLE,AQP1,C12orf25,MKI67,GPR175,BCL7B,OR4K5,DEFA6,HNF1B,C18orf23,SCOTIN,NCK1,AUP1,CHST5,FCN3,ZNF668,STAMBP,KRTAP2-4,OR6N1,CENPI,CCNF,OR4M1,PPM1G,IMP3,INHA,ANKRD33,STX1B,RNMT,PFN1,NUDT5,ADAMTS10,BCL11A,KCNIP2,FREM1,GNGT2,APC,XAB1,LOC390243,OR51B5,LOC203547,CSF1R,SHKBP1,NAT1,RUNDC3A,ZMYM6,GIT2,RPS16,IQCC,AANAT,TTTY10,PRKCA,STX4,ACSM2B,PRODH2,ORC5L,MAP3K9,NLRC3,CCDC24,PSPH,GNRH2,CDCA8,MPST |
| Colon | 172 | SLMAP,MEF2D,ZNF646,SH2D6,DDB1,PRKCG,PACSIN2,LCE3E,TRIM33,HCN1,CASP6,EIF3B,MED29,MAPKAPK2,ELOVL5,ARHGAP17,MANBAL,CDC14A,GTPBP1,SFRS1,ZC3H7A,SYT2,EVI2A,SMARCB1,FZD2,RNF34,USP24,PIK3C3,C11orf72,SEC23IP,ZZEF1,PTGES3,SCN4A,CNTN2,PSPN,EFHA2,SPRY2,SCYE1,ATP2C1,CHRNB4,AHCYL1,PDCD7,ABCB1,C17orf53,UBE2N,DYRK2,C6orf27,MTHFD1,MLL3,NALCN,LARP1,IL8,RTN4RL2,ABCE1,FLJ20309,MARVELD3,MCM10,CHP,CYP2C9,C2orf24,CCNDBP1,RUNDC3B,CDK4,MAN2A1,SNRPG,MAT2B,GPR44,THEM4,OTOF,MRPL11,ENOPH1,MT1X,OR4D2,GTSE1,SPCS1,LDOC1L,RAB31,RCC1,C3orf37,CTCF,CLASP2,DPP6,PABPN1,OLFM2,ZDHHC17,PTPRK,CRADD,C2orf18,BUB3,PIK3R4,DNAJC14,FOSL2,BMF,C11orf59,NIP7,RCOR1,TUBB2A,SAP130,KIAA0247,UVRAG,PPP5C,SPPL2A,FAM102A,ACAD9,HNRNPA2B1,NKX2-3,MAML1,TYK2,ACOT11,UNC5C,CIC,HNRPA3,KIAA1542,STAB1,ZKSCAN5,CAMK2A,PRODH,NPBWR1,SF3B1,BCL2L1,CEBPG,CCT3,MCM2,EGLN2,DHX29,NUMB,MAP3K4,CYLD,MED15,MLF2,MED9,RRM1,ERCC6,RC3H2,DPP8,DDX1,ITFG1,RNASEH2A,VPS54,SSSCA1,SFTPC,C14orf43,CTBP2,RAB40C,SETD1A,FIZ1,MARK4,CDC23,DYRK1A,SLC38A1,SYNJ1,C6orf106,MGC14327,KIAA0226,POFUT1,PRKACA,C10orf18,F2RL3,CENPC1,TOMM40,TLL1,MFSD5,FZD1,CBLC,FAM120B,TSHB,SUPT7L,CTTNBP2NL,MGRN1,GAB1,DRG2,FANCG |
| Colorectal | 179 | HPSE2,ERBB4,TACC2,CYP11A1,DIO3,VAMP8,SLC7A6,RLBP1,INCA,MATR3,TRIB3,USP47,FBXO42,KCNK3,CLCNKA,SPECC1L,KEL,FAM86C,UTF1,BCAN,ASB8,C19orf10,IKZF1,C6orf81,METTL2B,VAMP1,SLC25A5,CSTF3,OPHN1,KIF11,PSMB6,TNFRSF10A,CCT2,GABRR2,FTMT,DCLK1,FAM40A,RAB14,ZNHIT2,CYP1A1,EIF3EIP,NECAP2,WDR20,SLC6A18,PCCB,TAPBP,PJA2,ENAH,NYX,USP9X,KIAA1787,CATSPER1,CAMK2B,ZNF354C,JPH2,ANK2,HARS,RER1,OR9G4,MYCT1,PANK1,ROBO1,LOR,SDHD,SPEN,REV1,GNA15,ANKRD17,CLPS,CDC42,NT5E,SIGLEC1,EDF1,ARRB1,KIAA0406,TRIM69,OBSCN,LENG8,CAMK1G,LIMS1,SLC16A11,SF3B5,NRXN1,S100A8,PTPN9,UCK1,LEMD3,HCN3,KLK9,PSMA5,ZC3H14,SIGLEC6,CKS2,MYST1,AJAP1,SSPO,ZNF213,BTBD3,PLXNA2,SHE,SLC22A17,ERCC4,TFB2M,MAP3K7IP2,MPHOSPH10,SLC30A8,CDK5RAP1,BIRC6,PACSIN1,EIF4A1,PRIMA1,KIF17,FZD5,POLE2,SMARCA4,ALMS1L,PFDN1,HADH,UBOX5,ST8SIA3,CD63,TOP2A,NOX5,LDB2,SPINT1,NFE2L1,MAP3K12,PRX,IKZF4,DNM2,PPAP2B,PLXNB2,DGCR14,SFRS4,NSFL1C,ABCG5,PA2G4,SAP30BP,RIC8B,GPR17,LIN37,PANK2,MNT,SLC10A5,C14orf139,RAB11A,TMEM30A,BRPF3,C6orf85,GORASP2,C2orf25,DOK2,CIAPIN1,SOCS5,NFXL1,ACD,TUBB8,OR11A1,LARP4,MKL1,ANKRD32,SYNPO,HLA-C,ACOX2,CYP4F11,TA-NFKBH,ZFAND2B,UBE2D2,CSNK2A1,AMD1,SF3B3,PITPNB,NR1D1,ANXA11,NUSAP1,TTC26,FNBP1,AFF3,SLC44A4 |
| Rectum | 200 | DPM2,INSL3,EIF2B5,SKIP,C11orf47,CHD8,TLN1,ATXN7L1,ADAM7,UNC5A,RAB8B,PSG8,LRFN5,ODF1,PTOV1,NEK4,ASB10,RMND5B,PLEKHC1,EVC2,C9orf27,TUBB2C,ALG2,PRKCI,GRIPAP1,AIPL1,RACGAP1,THADA,DEFB119,TMCO2,ADORA2A,CNOT1,DDX18,KIAA0515,TEX261,MIOX,KIAA0317,ACADL,BPIL2,C2orf34,DTX1,KHK,EFNB3,IL5RA,DDX24,SETD8,PCDHGC5,GABBR1,ABCA8,RBM9,SNRPE,SLC35F1,LCK,RAF1,HNRNPA0,UQCRFS1,KLHL10,MEIS1,CRYBB3,NOP5/NOP58,SPACA1,RARS2,SERPINB13,C4orf8,KRTAP13-4,XKR5,ZNF414,C9orf140,OR8K3,ITPR1,C19orf29,NAP1L4,MIDN,BMP15,EAF1,ATXN2,APTX,CYP2A13,ESRRB,FASTKD2,CCKBR,PAK1IP1,IL3,CES7,EPC1,LELP1,C21orf2,RPL24,SLC14A2,ALDOA,DIS3L2,C11orf64,FBXO7,UPF1,GPR26,KIAA0372,TMEM16D,OR4K1,KIAA0831,RFWD3,NUP98,PRPF4,DLG2,EIF5B,XPO6,IRX6,PRIM1,STX17,SCAMP4,CA4,TSKU,MLLT6,PH-4,TOX4,NDST2,PRKD2,MBNL1,OR1A1,OTOS,NAT2,PALMD,BRUNOL5,CRYBB1,FOXI1,C1orf14,ACTL7A,C8orf31,SART1,APCS,CTDSP1,GABRR1,OR2B3,OR1C1,MCM4,OTOP3,KBTBD4,CHIA,SF4,ERLIN1,HBB,CCDC96,ZNF205,PCCA,ZNF143,GBF1,CCDC88C,BAI2,GRM7,CYP2C8,GLI2,PDCD11,TMEM41A,OR1N1,TBC1D10B,BAG5,MBD3,ATP6V0A1,PAK4,LSM2,OR1A2,RND2,RAD21,MICAL1,OR52N2,POP5,RARS,TIMM17B,COX4I1,RPS25,C2orf47,TTC9C,DRD1IP,RAB18,DEXI,PDSS2,MRPL36,NUP43,EPS15L1,RBM25,KPNA6,CYBRD1,PEAR1,HAP1,AP4E1,NRD1,ESR2,WAC,SNAP91,IL1R1,LOC652968,MAD2L2,VRK1,WDR23,LRRC3B,CFD,TXNDC6,FCHSD1,HLX,SAFB,PUM1 |
| Ovarian | 228 | ELMO2,PAX7,ADRA2C,PROZ,IGF2BP3,CTNNBL1,AQP9,CHRM5,OR9I1,SLC25A14,RHOT1,LHB,SMPD4,DEFB1,SLC22A18,SIGLEC9,FOXP3,CIAO1,TTLL6,GPR55,TAS2R39,OR52E4,DPH3,OR6M1,PAFAH1B1,GSG2,CYP21A2,RBM4,OTUD3,NEIL3,TRIM72,SLC2A3,PSMD3,RHEB,SIKE,C17orf79,TNXB,SUPT5H,LOC51145,DEFT1P,SNF1LK2,NNAT,MRPL28,CCT8,SYPL2,OR10G7,RAI14,CACNB1,SEC11C,FAM47B,C1orf111,LSG1,MARK3,ANTXR2,RPS13,ALX3,HCFC1,PRG3,RBM6,ATP1A1,NKG7,HP,RPL10,TAGAP,TRAF2,KIAA1524,PARG,HAPLN4,WNT3A,CKS1B,ZDHHC4,CCT4,KSR1,C9orf7,OAS3,G6PC2,C20orf77,CTSS,CDGAP,PCBP1,CCDC132,PRUNE,RBM26,LOC407835,MAP2K2,KCNH4,HIST1H2BB,DDI2,TFE3,DCT,PPP2CB,SIRPG,DKFZp761B107,STX11,CA9,OR10J1,TNK2,CBX1,MAP4K3,CTNNB1,CIDEA,GCM1,TCFL5,SFRS10,HNRPLL,PSMD2,ZNF740,CLDN10,EIF3F,ARS2,RGR,CCDC14,NEDD1,MYC,LAMA3,DIS3,PDXP,PLA2G10,PSMC1,MS4A6E,EIF4G1,AGPAT6,GPR31,FLJ12716,IHH,CD37,ABCG2,KRTAP12-1,MEN1,MIXL1,PPP1R12B,ASPM,DVL3,SLC2A14,ENDOGL1,C12orf41,SMURF1,CSTF2,AGTRL1,GDAP2,CRH,RNF167,PGF,API5,PLA2G1B,ADRA2B,PPM1B,TNFSF11,GPR42,GALR3,FLJ43826,FAM128B,LDHAL6B,MTMR4,USP21,CFL1,TRHR,OR10W1,IARS,ATXN7L3,MUC17,PRTN3,SP2,PLA2G4F,LILRA1,SLC24A4,PDHA2,HIST1H3C,TRIM44,PARL,CABP2,RNF133,P2RX1,TREH,RAB5C,FGF10,FAM3A,RAD51AP1,H2AFV,CYP17A1,TLR4,UBQLN1,UNQ1940,FFAR2,SEC11A,PSMD1,OR10H3,PRRG3,PSORS1C2,CIT,B3GNT6,SCAF1,RPS6KA1,C15orf28,CUL2,HIST1H3D,RP1L1,CHSY-2,PITX3,GSTA5,MFAP1,ADNP,ZNF19,C7orf28B,IFNB1,KISS1,GPX1,CRISPLD2,SNAI2,DDX3X,SAMD4B,GLP2R,USP39,POLR3D,SUMO1P1,FCRL1,DKFZp564N2472,FDPS,CDH5,CSF2RB,ZNF645,VPS13B,CPSF2,HIST2H3C,OR52K2,MLXIP,ADIPOQ,ZNF638 |
| Glioblastoma | 73 | DECR1,IRAK1,CTNND2,EP300,SEMA4D,GNAI2,ACTR8,RFWD2,MRVI1,DDB2,STARD5,EXOSC2,TAS1R3,ADI1,AGPAT3,HVCN1,FGD4,KCNE3,HMG1L1,TAP1,CHI3L1,LPPR2,PCSK6,OXCT1,POLD2,EMID1,FURIN,LENG4,JAK2,HSPA6,ARSB,C1orf159,KRT83,POMGNT1,TCEB3,PDCD6IP,CLIC5,SLC35B3,C17orf87,TIMP1,WWP1,KCNQ1,L1CAM,NPAS2,MAN1A1,HIST1H1B,SMC2,DNAJC5G,TAP2,GPR4,RPL15,CACNA1A,CASC5,CENTA2,CNGA4,CHMP1A,FCRL4,KRT7,NEDD8,STX10,UBTD2,CSNK1G3,LSM1,CANX,APOBEC3G,PLVAP,EPAS1,FPRL2,CEBPB,RPS6KA3,KIAA0825,PTGIS,DARS2 |
| Glioma | 69 | TNIP3,HPN,TICAM1,GABARAP,BBC3,TMEM86A,SMAD3,HIF3A,HRH4,PI4KA,SCIN,NUP37,PPAP2C,CACNG7,ITGA1,HP1BP3,RNF103,TMPO,SLC22A1,IKBKG,GPKOW,CD27,ATP6V1A,IGF2AS,MSR1,PASD1,SSTR3,EEF2K,SOCS3,NR3C2,PRKAA2,DCST1,ADRBK1,LRRC32,SLC35F5,BTN1A1,CYP1A2,HRBL,C2orf29,IGF2,TNRC4,ZC3HC1,GLTSCR1,FMO3,MGC29506,GRIK5,WNT10A,F10,CD300LB,C6orf153,SYNE1,HIST1H2AH,VCAN,DLD,AMY2B,PRM2,TPRKB,C1orf127,TMEM48,PLA2G4B,LIMK1,HMGA1,CPN2,APBB1IP,TRIM25,HEXA,PLAUR,PDE3B,HOPX |

**Supplementary Table 2:** Significant pathways for the case of common genes between the seven cancer types

| **Pathways** | **P-value** | **Genes** |
| --- | --- | --- |
| Alcoholism | 3.65375E-20 | HIST1H4K;HIST1H4L;MAOA;GNG12;HIST1H4A;NRAS;HIST1H4B;HIST1H4H;CALM3;HIST1H4J;HIST1H4C;CALM1;CALM2;HIST1H4D;HRAS;HIST1H4E;HIST1H4F |
| Viral carcinogenesis | 1.25315E-17 | CDKN1A;CDKN1B;HIST1H4K;HIST1H4L;HIST1H4A;NRAS;HIST1H4B;CASP3;HIST1H4H;HIST1H4J;HIST1H4C;HIST1H4D;TP53;HRAS;HIST1H4E;HIST1H4F |
| Glioma | 2.17014E-14 | NRAS;CDKN1A;EGF;AKT2;AKT1;CALM3;CALM1;CALM2;TP53;HRAS |
| Pathways in cancer | 2.47394E-14 | CDKN1A;CDKN1B;TGFB1;EGF;ADCY8;GNG12;NRAS;FGF9;AKT2;CASP3;AKT1;PLCB1;BID;WNT1;TP53;HRAS;FGF23 |
| Ras signaling pathway | 4.33766E-14 | NGFR;EGF;RASAL1;GNG12;NRAS;FGF9;AKT2;AKT1;CALM3;CALM1;CALM2;HRAS;PAK2;FGF23 |
| Rap1 signaling pathway | 3.8707E-13 | NGFR;EGF;ADCY8;NRAS;FGF9;AKT2;AKT1;CALM3;CALM1;PLCB1;CALM2;HRAS;FGF23 |
| FoxO signaling pathway | 1.14593E-12 | NRAS;CCNB1;CDKN1A;TGFB1;CDKN1B;EGF;AKT2;PLK1;AKT1;HRAS;PRKAG3 |
| Estrogen signaling pathway | 1.72083E-12 | NRAS;AKT2;AKT1;CALM3;CALM1;ADCY8;PLCB1;CALM2;HRAS;HSPA1A |
| Melanoma | 2.89954E-12 | NRAS;CDKN1A;FGF9;EGF;AKT2;AKT1;TP53;FGF23;HRAS |
| MAPK signaling pathway | 4.31369E-12 | TGFB1;EGF;GNG12;NRAS;FGF9;AKT2;CASP3;AKT1;TP53;HRAS;PAK2;FGF23;HSPA1A |
| PI3K-Akt signaling pathway | 1.09848E-11 | NGFR;CDKN1A;CDKN1B;EGF;GNG12;NRAS;FGF9;PPP2R1B;PPP2R1A;AKT2;AKT1;TP53;HRAS;FGF23 |
| Dopaminergic synapse | 2.50197E-11 | PPP2R1B;PPP2R1A;MAOA;AKT2;AKT1;CALM3;CALM1;GNG12;PLCB1;CALM2 |
| Systemic lupus erythematosus | 3.94146E-11 | HIST1H4A;HIST1H4B;HIST1H4K;HIST1H4L;HIST1H4H;HIST1H4J;HIST1H4C;HIST1H4D;HIST1H4E;HIST1H4F |
| AGE-RAGE signaling pathway in diabetic complications | 7.48593E-11 | NRAS;TGFB1;CDKN1B;AKT2;CASP3;AKT1;PLCB1;AGER;HRAS |
| Hepatitis B | 8.59375E-11 | NRAS;CDKN1A;TGFB1;PCNA;CDKN1B;AKT2;CASP3;AKT1;TP53;HRAS |
| Chronic myeloid leukemia | 1.67261E-10 | NRAS;CDKN1A;TGFB1;CDKN1B;AKT2;AKT1;TP53;HRAS |
| Neurotrophin signaling pathway | 3.5572E-10 | NGFR;NRAS;AKT2;AKT1;CALM3;CALM1;CALM2;TP53;HRAS |
| Sphingolipid signaling pathway | 3.5572E-10 | NRAS;PPP2R1B;PPP2R1A;AKT2;AKT1;PLCB1;BID;TP53;HRAS |
| ErbB signaling pathway | 6.99508E-10 | NRAS;CDKN1A;CDKN1B;EGF;AKT2;AKT1;PAK2;HRAS |
| Prostate cancer | 8.40797E-10 | NRAS;CDKN1A;CDKN1B;EGF;AKT2;AKT1;TP53;HRAS |
| Hepatitis C | 8.93146E-10 | NRAS;CDKN1A;PPP2R1B;EGF;PPP2R1A;AKT2;AKT1;TP53;HRAS |
| Insulin signaling pathway | 1.32284E-09 | NRAS;PRKAR2B;AKT2;AKT1;CALM3;CALM1;CALM2;HRAS;PRKAG3 |
| Melanogenesis | 2.14907E-09 | NRAS;CALM3;CALM1;ADCY8;WNT1;PLCB1;CALM2;HRAS |
| Adrenergic signaling in cardiomyocytes | 2.30716E-09 | PPP2R1B;PPP2R1A;AKT2;AKT1;CALM3;CALM1;ADCY8;PLCB1;CALM2 |
| Longevity regulating pathway - multiple species | 2.50789E-09 | NRAS;AKT2;AKT1;ADCY8;HRAS;PRKAG3;HSPA1A |
| Long-term potentiation | 3.12671E-09 | NRAS;CALM3;CALM1;ADCY8;PLCB1;CALM2;HRAS |
| Oxytocin signaling pathway | 4.10781E-09 | NRAS;CDKN1A;CALM3;CALM1;ADCY8;PLCB1;CALM2;HRAS;PRKAG3 |
| Oocyte meiosis | 1.11613E-08 | CCNB1;PPP2R1B;PPP2R1A;PLK1;CALM3;CALM1;ADCY8;CALM2 |
| HTLV-I infection | 2.15551E-08 | CDKN1A;NRAS;TGFB1;PCNA;AKT2;AKT1;ADCY8;WNT1;TP53;HRAS |
| Endometrial cancer | 2.64856E-08 | NRAS;EGF;AKT2;AKT1;TP53;HRAS |
| GnRH signaling pathway | 3.03287E-08 | NRAS;CALM3;CALM1;ADCY8;PLCB1;CALM2;HRAS |
| Proteoglycans in cancer | 3.64692E-08 | CDKN1A;NRAS;TGFB1;AKT2;CASP3;AKT1;WNT1;TP53;HRAS |
| Longevity regulating pathway - mammal | 3.80381E-08 | NRAS;AKT2;AKT1;ADCY8;TP53;HRAS;PRKAG3 |
| Non-small cell lung cancer | 4.17788E-08 | NRAS;EGF;AKT2;AKT1;TP53;HRAS |
| Glucagon signaling pathway | 6.2693E-08 | AKT2;AKT1;CALM3;CALM1;PLCB1;CALM2;PRKAG3 |
| Renal cell carcinoma | 1.13747E-07 | NRAS;TGFB1;AKT2;AKT1;PAK2;HRAS |
| Cholinergic synapse | 1.20427E-07 | NRAS;AKT2;AKT1;ADCY8;GNG12;PLCB1;HRAS |
| DNA replication | 1.57388E-07 | RFC5;POLA1;RFC4;PCNA;RPA1 |
| Tuberculosis | 1.9836E-07 | TGFB1;AKT2;CASP3;AKT1;CALM3;CALM1;BID;CALM2 |
| Cell cycle | 2.57499E-07 | CCNB1;CDKN1A;TGFB1;PCNA;CDKN1B;PLK1;TP53 |
| Bladder cancer | 3.08652E-07 | CDKN1A;NRAS;EGF;TP53;HRAS |
| Tight junction | 5.59955E-07 | NRAS;PPP2R1B;PPP2R1A;AKT2;AKT1;MYH11;HRAS |
| Apoptosis | 5.87802E-07 | NRAS;AKT2;CASP3;AKT1;BID;TP53;HRAS |
| Nucleotide excision repair | 6.21592E-07 | RFC5;RFC4;PCNA;ERCC2;RPA1 |
| Gap junction | 6.38695E-07 | NRAS;GJA1;EGF;ADCY8;PLCB1;HRAS |
| Phospholipase D signaling pathway | 7.1112E-07 | NRAS;EGF;AKT2;AKT1;ADCY8;PLCB1;HRAS |
| Circadian entrainment | 1.0048E-06 | CALM3;GNG12;CALM1;ADCY8;PLCB1;CALM2 |
| Mismatch repair | 1.15858E-06 | RFC5;PCNA;RFC4;RPA1 |
| T cell receptor signaling pathway | 1.71151E-06 | CD4;NRAS;AKT2;AKT1;PAK2;HRAS |
| Chagas disease (American trypanosomiasis) | 1.71151E-06 | TGFB1;PPP2R1B;PPP2R1A;AKT2;AKT1;PLCB1 |
| cGMP-PKG signaling pathway | 1.92231E-06 | AKT2;AKT1;CALM3;CALM1;ADCY8;PLCB1;CALM2 |
| Long-term depression | 2.13659E-06 | NRAS;PPP2R1B;PPP2R1A;PLCB1;HRAS |
| Colorectal cancer | 2.51784E-06 | TGFB1;AKT2;CASP3;AKT1;TP53 |
| Serotonergic synapse | 2.63991E-06 | NRAS;MAOA;CASP3;GNG12;PLCB1;HRAS |
| Pancreatic cancer | 3.43988E-06 | TGFB1;EGF;AKT2;AKT1;TP53 |
| Thyroid hormone signaling pathway | 3.57669E-06 | NRAS;AKT2;AKT1;PLCB1;TP53;HRAS |
| Central carbon metabolism in cancer | 3.70724E-06 | NRAS;AKT2;AKT1;TP53;HRAS |
| Amphetamine addiction | 3.70724E-06 | MAOA;CALM3;CALM1;CALM2;STX1A |
| Vascular smooth muscle contraction | 3.94316E-06 | CALM3;MYH11;CALM1;ADCY8;PLCB1;CALM2 |
| Chemokine signaling pathway | 4.07111E-06 | NRAS;AKT2;AKT1;ADCY8;GNG12;PLCB1;HRAS |
| p53 signaling pathway | 4.29079E-06 | CDKN1A;CCNB1;CASP3;BID;TP53 |
| AMPK signaling pathway | 4.76791E-06 | PPP2R1B;PPP2R1A;AKT2;AKT1;CD36;PRKAG3 |
| Gastric acid secretion | 6.06757E-06 | CALM3;CALM1;ADCY8;PLCB1;CALM2 |
| Aldosterone synthesis and secretion | 9.46965E-06 | CALM3;CALM1;ADCY8;PLCB1;CALM2 |
| Regulation of actin cytoskeleton | 9.85477E-06 | NRAS;FGF9;EGF;GNG12;HRAS;PAK2;FGF23 |
| Non-alcoholic fatty liver disease (NAFLD) | 1.47627E-05 | TGFB1;AKT2;CASP3;AKT1;BID;PRKAG3 |
| Salivary secretion | 1.50127E-05 | CALM3;CALM1;ADCY8;PLCB1;CALM2 |
| Progesterone-mediated oocyte maturation | 2.39719E-05 | CCNB1;AKT2;PLK1;AKT1;ADCY8 |
| Inflammatory mediator regulation of TRP channels | 2.39719E-05 | CALM3;CALM1;ADCY8;PLCB1;CALM2 |
| Alzheimer's disease | 2.7002E-05 | CASP3;CALM3;CALM1;PLCB1;BID;CALM2 |
| Choline metabolism in cancer | 2.77341E-05 | NRAS;EGF;AKT2;AKT1;HRAS |
| HIF-1 signaling pathway | 3.04864E-05 | CDKN1A;CDKN1B;EGF;AKT2;AKT1 |
| Acute myeloid leukemia | 4.72499E-05 | NRAS;AKT2;AKT1;HRAS |
| Toxoplasmosis | 5.85112E-05 | TGFB1;AKT2;CASP3;AKT1;HSPA1A |
| VEGF signaling pathway | 6.17686E-05 | NRAS;AKT2;AKT1;HRAS |
| cAMP signaling pathway | 6.9494E-05 | AKT2;AKT1;CALM3;CALM1;ADCY8;CALM2 |
| Renin secretion | 7.46136E-05 | CALM3;CALM1;PLCB1;CALM2 |
| Epstein-Barr virus infection | 7.54857E-05 | CDKN1A;CDKN1B;AKT2;AKT1;TP53;HSPA1A |
| Fc epsilon RI signaling pathway | 9.46333E-05 | NRAS;AKT2;AKT1;HRAS |
| Adipocytokine signaling pathway | 0.000105986 | AKT2;AKT1;CD36;PRKAG3 |
| Phototransduction | 0.000113076 | CALM3;CALM1;CALM2 |
| Measles | 0.000114658 | CDKN1B;AKT2;AKT1;TP53;HSPA1A |
| Prolactin signaling pathway | 0.000118296 | NRAS;AKT2;AKT1;HRAS |
| B cell receptor signaling pathway | 0.000124825 | NRAS;AKT2;AKT1;HRAS |
| Pertussis | 0.00013866 | CASP3;CALM3;CALM1;CALM2 |
| Signaling pathways regulating pluripotency of stem cells | 0.000140443 | NRAS;AKT2;AKT1;WNT1;HRAS |
| Thyroid cancer | 0.000140551 | NRAS;TP53;HRAS |
| Small cell lung cancer | 0.000235351 | CDKN1B;AKT2;AKT1;TP53 |
| mRNA surveillance pathway | 0.000292342 | NCBP1;PPP2R1B;NCBP2;PPP2R1A |
| Phosphatidylinositol signaling system | 0.00038792 | CALM3;CALM1;PLCB1;CALM2 |
| Calcium signaling pathway | 0.000421219 | CALM3;CALM1;ADCY8;PLCB1;CALM2 |
| Insulin resistance | 0.000580429 | AKT2;AKT1;CD36;PRKAG3 |
| MicroRNAs in cancer | 0.000602463 | CDKN1A;NRAS;CDKN1B;CASP3;TP53;HRAS |
| Focal adhesion | 0.000710963 | EGF;AKT2;AKT1;HRAS;PAK2 |
| Amyotrophic lateral sclerosis (ALS) | 0.000758101 | CASP3;BID;TP53 |
| Platelet activation | 0.00088568 | AKT2;AKT1;ADCY8;PLCB1 |
| Regulation of lipolysis in adipocytes | 0.000996569 | AKT2;AKT1;ADCY8 |
| Natural killer cell mediated cytotoxicity | 0.001289664 | NRAS;CASP3;BID;HRAS |
| Hippo signaling pathway | 0.002040619 | TGFB1;PPP2R1B;PPP2R1A;WNT1 |
| Jak-STAT signaling pathway | 0.002293482 | CDKN1A;AKT2;AKT1;HRAS |
| RNA transport | 0.003115025 | NCBP1;NCBP2;NUP88;AAAS |
| TGF-beta signaling pathway | 0.003194535 | TGFB1;PPP2R1B;PPP2R1A |
| Insulin secretion | 0.003303176 | ADCY8;PLCB1;STX1A |
| Transcriptional misregulation in cancer | 0.003664829 | NGFR;CDKN1A;CDKN1B;TP53 |
| Amoebiasis | 0.005209802 | TGFB1;CASP3;PLCB1 |
| Retrograde endocannabinoid signaling | 0.005355968 | ADCY8;GNG12;PLCB1 |
| TNF signaling pathway | 0.006783049 | AKT2;CASP3;AKT1 |
| Glutamatergic synapse | 0.007483013 | ADCY8;GNG12;PLCB1 |
| Axon guidance | 0.010045682 | NRAS;PAK2;HRAS |
| Carbohydrate digestion and absorption | 0.010848866 | AKT2;AKT1 |
| Osteoclast differentiation | 0.011151001 | TGFB1;AKT2;AKT1 |
| Spliceosome | 0.011612047 | NCBP1;NCBP2;HSPA1A |
| Malaria | 0.012771973 | TGFB1;CD36 |
| Endocytosis | 0.01292516 | TGFB1;EGF;HRAS;HSPA1A |
| Wnt signaling pathway | 0.013565407 | WNT1;PLCB1;TP53 |
| Legionellosis | 0.015912445 | CASP3;HSPA1A |
| Basal cell carcinoma | 0.015912445 | WNT1;TP53 |
| Viral myocarditis | 0.018170716 | CASP3;BID |
| mTOR signaling pathway | 0.018755292 | AKT2;AKT1 |
| Influenza A | 0.023504026 | AKT2;AKT1;HSPA1A |
| PPAR signaling pathway | 0.024363894 | UBC;CD36 |
| Thyroid hormone synthesis | 0.025692565 | ADCY8;PLCB1 |
| Antigen processing and presentation | 0.029850298 | CD4;HSPA1A |
| Huntington's disease | 0.03021533 | CASP3;PLCB1;TP53 |
| Hypertrophic cardiomyopathy (HCM) | 0.034256169 | TGFB1;PRKAG3 |
| Hematopoietic cell lineage | 0.038108739 | CD4;CD36 |
| GABAergic synapse | 0.038108739 | GNG12;ADCY8 |
| Dilated cardiomyopathy | 0.039694222 | TGFB1;ADCY8 |
| Morphine addiction | 0.04049628 | GNG12;ADCY8 |
| Fc gamma R-mediated phagocytosis | 0.04211877 | AKT2;AKT1 |
| Pancreatic secretion | 0.044597672 | ADCY8;PLCB1 |

**Supplementary Table 3:** Significant pathways for the case of the exclusive breast cancer genes

| Pathways | P-value | Genes |
| --- | --- | --- |
| Hepatitis B | 0.000223575 | TGFB2;CASP8;APAF1;VDAC3;PTEN;STAT6;PRKCA;KRAS |
| Pathways in cancer | 0.000498461 | CSF1R;TGFB2;ITGA2;PTEN;PRKCA;STK4;GNG2;GNGT2;CASP8;APC;ERBB2;RARA;KRAS |
| Cholinergic synapse | 0.001469101 | CHRNB2;CHRM1;GNG2;GNGT2;PRKCA;KRAS |
| Serotonergic synapse | 0.001538208 | DDC;GNG2;GNGT2;ALOX15;PRKCA;KRAS |
| Endometrial cancer | 0.002593708 | APC;ERBB2;PTEN;KRAS |
| Metabolic pathways | 0.002657247 | DDC;ALOX15;ACSM2A;ACSM2B;PAPSS2;TPO;AANAT;HSD17B1;POLR2D;ATP6V0A2;POLR2F;HMGCS2;POLE;PSPH;MPST;AGL;CEL;PRODH2;GPAM;NAT1;POLR1A;PNPLA3;ALPI;SUCLG1;PI4K2A |
| Non-small cell lung cancer | 0.003400258 | ERBB2;PRKCA;KRAS;STK4 |
| Purine metabolism | 0.003487228 | NPR1;POLR1A;POLR2D;POLR2F;NUDT5;POLE;PAPSS2 |
| Butanoate metabolism | 0.003556293 | ACSM2A;HMGCS2;ACSM2B |
| Sulfur metabolism | 0.005159528 | MPST;PAPSS2 |
| RNA polymerase | 0.005212813 | POLR1A;POLR2D;POLR2F |
| p53 signaling pathway | 0.00716192 | CASP8;APAF1;PTEN;PMAIP1 |
| Glutamatergic synapse | 0.008737493 | GNG2;GNGT2;PRKCA;GRIK2;SHANK3 |
| Dopaminergic synapse | 0.014359944 | DDC;GNG2;GNGT2;PRKCA;DRD3 |
| Phenylalanine metabolism | 0.014821053 | DDC;GLYAT |
| ErbB signaling pathway | 0.015826123 | ERBB2;PRKCA;KRAS;NCK1 |
| GABAergic synapse | 0.016440054 | GNGT2;GNG2;PRKCA;SLC6A1 |
| Apoptosis | 0.019758989 | CASP8;TUBA1A;APAF1;PMAIP1;KRAS |
| Huntington's disease | 0.020559165 | CASP8;APAF1;POLR2D;VDAC3;POLR2F;COX7A1 |
| Legionellosis | 0.022920154 | CASP8;APAF1;CXCL3 |
| Pathogenic Escherichia coli infection | 0.022920154 | TUBA1A;PRKCA;NCK1 |
| HTLV-I infection | 0.024931009 | CDC20;TGFB2;APC;VDAC3;KRAS;POLE;ANAPC2 |
| Glycerolipid metabolism | 0.027510026 | GPAM;PNPLA3;CEL |
| Pyrimidine metabolism | 0.029201373 | POLR1A;POLR2D;POLR2F;POLE |
| Colorectal cancer | 0.031249535 | TGFB2;APC;KRAS |
| Neuroactive ligand-receptor interaction | 0.034719421 | CHRNB2;CHRM1;AVPR1B;CHRNE;TAAR2;GRIK2;DRD3 |
| Glioma | 0.035241778 | PTEN;PRKCA;KRAS |
| PI3K-Akt signaling pathway | 0.036270841 | CSF1R;GNG2;GNGT2;CHRM1;ITGA2;PTEN;PRKCA;KRAS |
| Pancreatic cancer | 0.036628268 | TGFB2;ERBB2;KRAS |
| Central carbon metabolism in cancer | 0.038042448 | ERBB2;PTEN;KRAS |
| Ras signaling pathway | 0.04070334 | CSF1R;GNG2;GNGT2;PRKCA;KRAS;STK4 |
| MicroRNAs in cancer | 0.047478829 | TGFB2;APC;ERBB2;PTEN;PRKCA;KIF23;KRAS |
| Calcium signaling pathway | 0.049863139 | CHRM1;AVPR1B;ERBB2;VDAC3;PRKCA |

**Supplementary Table 4:** Significant pathways for the case of the exclusive colon cancer genes

| Pathways | P-value | Genes |
| --- | --- | --- |
| Wnt signaling pathway | 0.001446927 | PRKCG;FZD1;FZD2;CTBP2;CAMK2A;PRKACA |
| Melanogenesis | 0.001730122 | PRKCG;FZD1;FZD2;CAMK2A;PRKACA |
| Proteoglycans in cancer | 0.001909604 | PRKCG;FZD1;FZD2;CAMK2A;GAB1;CBLC;PRKACA |
| Pathways in cancer | 0.00235737 | PRKCG;FZD1;FZD2;EGLN2;CTBP2;CDK4;CBLC;PRKACA;BCL2L1;F2RL3 |
| Chronic myeloid leukemia | 0.003624991 | CTBP2;CDK4;CBLC;BCL2L1 |
| Cell cycle | 0.004389254 | CDC23;CDK4;BUB3;CDC14A;MCM2 |
| Ubiquitin mediated proteolysis | 0.006664038 | DDB1;CDC23;MGRN1;UBE2N;CBLC |
| ErbB signaling pathway | 0.006757849 | PRKCG;CAMK2A;GAB1;CBLC |
| Cysteine and methionine metabolism | 0.006805942 | AHCYL1;MAT2B;ENOPH1 |
| HTLV-I infection | 0.007079838 | FZD1;CDC23;FZD2;CDK4;BUB3;PRKACA;BCL2L1 |
| Notch signaling pathway | 0.008141602 | CTBP2;MAML1;NUMB |
| Cholinergic synapse | 0.01552806 | PRKCG;CHRNB4;CAMK2A;PRKACA |
| Glioma | 0.018497274 | PRKCG;CDK4;CAMK2A |
| Long-term potentiation | 0.019258363 | PRKCG;CAMK2A;PRKACA |
| Amphetamine addiction | 0.020036583 | PRKCG;CAMK2A;PRKACA |
| Thyroid hormone synthesis | 0.023320976 | PRKCG;PRKACA;TSHB |
| Gastric acid secretion | 0.025964391 | PRKCG;CAMK2A;PRKACA |
| Aldosterone synthesis and secretion | 0.032730413 | PRKCG;CAMK2A;PRKACA |
| Insulin secretion | 0.036969437 | PRKCG;CAMK2A;PRKACA |
| DNA replication | 0.0382699 | RNASEH2A;MCM2 |
| GABAergic synapse | 0.04032437 | PRKCG;SLC38A1;PRKACA |
| Gap junction | 0.04032437 | PRKCG;TUBB2A;PRKACA |
| GnRH signaling pathway | 0.043828122 | CAMK2A;PRKACA;MAP3K4 |
| Regulation of autophagy | 0.044270019 | PIK3R4;PIK3C3 |
| Circadian entrainment | 0.048728114 | PRKCG;CAMK2A;PRKACA |

**Supplementary Table 5:** Significant pathways for the case of the exclusive colorectal cancer genes

| Pathways | P-value | Genes |
| --- | --- | --- |
| Proteoglycans in cancer | 0.002391426 | CDC42;CAMK2B;CD63;HPSE2;FZD5;ERBB4;ANK2 |
| Fat digestion and absorption | 0.005854851 | ABCG5;CLPS;PPAP2B |
| Aldosterone synthesis and secretion | 0.006043296 | CAMK2B;CYP11A1;KCNK3;CAMK1G |
| Pantothenate and CoA biosynthesis | 0.011092326 | PANK2;PANK1 |
| Axon guidance | 0.027417018 | CDC42;PLXNA2;PLXNB2;ROBO1 |
| Endocytosis | 0.029351306 | CDC42;ERBB4;HLA-C;ARRB1;RAB11A;DNM2 |
| SNARE interactions in vesicular transport | 0.037056569 | VAMP8;VAMP1 |
| Tryptophan metabolism | 0.049769192 | CYP1A1;HADH |

**Supplementary Table 6:** Significant pathways for the case of the exclusive rectum genes

| Pathways | P-value | Genes |
| --- | --- | --- |
| RNA transport | 0.007768749 | EIF2B5;POP5;EIF5B;UPF1;NUP43;NUP98 |
| Spliceosome | 0.011243883 | PRPF4;SART1;RBM25;SNRPE;LSM2 |
| Estrogen signaling pathway | 0.017483524 | GABBR1;ITPR1;RAF1;ESR2 |
| Fructose and mannose metabolism | 0.040555749 | ALDOA;KHK |
| Caffeine metabolism | 0.049014754 | NAT2 |
| Chemical carcinogenesis | 0.049102279 | CYP2C8;CYP2A13;NAT2 |

**Supplementary Table 7:** Significant pathways for the case of the exclusive ovarian cancer genes

| Pathways | P-value | Genes |
| --- | --- | --- |
| Proteasome | 0.001561994 | PSMC1;PSMD2;PSMD3;PSMD1 |
| Ether lipid metabolism | 0.001699235 | PLA2G4F;PLA2G1B;PLA2G10;PAFAH1B1 |
| PI3K-Akt signaling pathway | 0.001926152 | PPP2CB;G6PC2;TNXB;MAP2K2;IFNB1;RHEB;MYC;LAMA3;TLR4;PGF;FGF10 |
| MAPK signaling pathway | 0.002699083 | CACNB1;PLA2G4F;PPM1B;MAP2K2;MYC;RPS6KA1;TRAF2;MAP4K3;FGF10 |
| alpha-Linolenic acid metabolism | 0.00279421 | PLA2G4F;PLA2G1B;PLA2G10 |
| Thyroid cancer | 0.004293049 | MAP2K2;MYC;CTNNB1 |
| Linoleic acid metabolism | 0.004293049 | PLA2G4F;PLA2G1B;PLA2G10 |
| Ras signaling pathway | 0.004627758 | PLA2G4F;RAB5C;MAP2K2;KSR1;PLA2G1B;PLA2G10;PGF;FGF10 |
| Transcriptional misregulation in cancer | 0.004671799 | PAX7;MYC;TFE3;HIST1H3C;HIST2H3C;HIST1H3D;MEN1 |
| Long-term depression | 0.004870074 | PPP2CB;PLA2G4F;MAP2K2;CRH |
| Arachidonic acid metabolism | 0.005473863 | PLA2G4F;GPX1;PLA2G1B;PLA2G10 |
| Melanogenesis | 0.005773918 | MAP2K2;DCT;WNT3A;DVL3;CTNNB1 |
| Pathways in cancer | 0.006055012 | MAP2K2;WNT3A;MYC;CUL2;LAMA3;DVL3;CTNNB1;TRAF2;PGF;FGF10;CKS1B |
| Hippo signaling pathway | 0.008281396 | PPP2CB;WNT3A;MYC;SNAI2;DVL3;CTNNB1 |
| Epstein-Barr virus infection | 0.008614521 | POLR3D;PSMC1;MYC;PSMD2;PSMD3;PSMD1;TRAF2 |
| Prolactin signaling pathway | 0.009244775 | MAP2K2;LHB;TNFSF11;CYP17A1 |
| Thyroid hormone signaling pathway | 0.011393092 | MAP2K2;RHEB;MYC;CTNNB1;ATP1A1 |
| Vascular smooth muscle contraction | 0.012186976 | PLA2G4F;MAP2K2;PLA2G1B;PLA2G10;PPP1R12B |
| Neuroactive ligand-receptor interaction | 0.014426554 | GALR3;P2RX1;LHB;CHRM5;GLP2R;ADRA2C;ADRA2B;TRHR |
| Small cell lung cancer | 0.016880955 | MYC;LAMA3;TRAF2;CKS1B |
| Alcoholism | 0.01693294 | CRH;H2AFV;HIST1H2BB;HIST1H3C;HIST2H3C;HIST1H3D |
| Osteoclast differentiation | 0.017746182 | IFNB1;SIRPG;TNFSF11;TRAF2;LILRA1 |
| Hepatitis C | 0.018273741 | CLDN10;PPP2CB;IFNB1;OAS3;TRAF2 |
| Ovarian steroidogenesis | 0.019341805 | PLA2G4F;LHB;CYP17A1 |
| Systemic lupus erythematosus | 0.01935969 | H2AFV;HIST1H2BB;HIST1H3C;HIST2H3C;HIST1H3D |
| Endometrial cancer | 0.021451967 | MAP2K2;MYC;CTNNB1 |
| Glycerophospholipid metabolism | 0.023398324 | AGPAT6;PLA2G4F;PLA2G1B;PLA2G10 |
| Signaling pathways regulating pluripotency of stem cells | 0.023491497 | MAP2K2;WNT3A;MYC;DVL3;CTNNB1 |
| Basal cell carcinoma | 0.024844087 | WNT3A;DVL3;CTNNB1 |
| Hepatitis B | 0.026089761 | MAP2K2;DDX3X;IFNB1;MYC;TLR4 |
| Protein export | 0.027971034 | SEC11A;SEC11C |
| Proteoglycans in cancer | 0.029171216 | MAP2K2;WNT3A;MYC;CTNNB1;PPP1R12B;TLR4 |
| HIF-1 signaling pathway | 0.030314875 | PDHA2;MAP2K2;CUL2;TLR4 |
| Central carbon metabolism in cancer | 0.041113834 | PDHA2;MAP2K2;MYC |
| Glycolysis / Gluconeogenesis | 0.041113834 | PDHA2;G6PC2;LDHAL6B |
| Adipocytokine signaling pathway | 0.045843522 | G6PC2;ADIPOQ;TRAF2 |
| RIG-I-like receptor signaling pathway | 0.045843522 | DDX3X;IFNB1;TRAF2 |
| Bile secretion | 0.047477434 | AQP9;ATP1A1;ABCG2 |
| Olfactory transduction | 0.049053731 | OR10J1;OR9I1;SLC24A4;OR10H3;OR52E4;OR10W1;OR6M1;OR10G7;OR52K2 |

**Supplementary Table 8:** Significant pathways for the case of the exclusive glioma genes

| Pathways | P-value | Genes |
| --- | --- | --- |
| Fc gamma R-mediated phagocytosis | 0.000300602 | SCIN;PLA2G4B;LIMK1;PPAP2C |
| Adipocytokine signaling pathway | 0.00182348 | SOCS3;PRKAA2;IKBKG |
| Oxytocin signaling pathway | 0.002175631 | CACNG7;PRKAA2;EEF2K;PLA2G4B |
| Hypertrophic cardiomyopathy (HCM) | 0.002964571 | CACNG7;PRKAA2;ITGA1 |
| NF-kappa B signaling pathway | 0.004085566 | TRIM25;IKBKG;TICAM1 |
| Linoleic acid metabolism | 0.004484449 | PLA2G4B;CYP1A2 |
| Choline metabolism in cancer | 0.005145071 | PLA2G4B;SLC22A1;PPAP2C |
| Chagas disease (American trypanosomiasis) | 0.005581083 | SMAD3;IKBKG;TICAM1 |
| Glutamatergic synapse | 0.007191761 | PLA2G4B;GRIK5;ADRBK1 |
| Regulation of autophagy | 0.008005072 | PRKAA2;GABARAP |
| Ether lipid metabolism | 0.01055388 | PLA2G4B;PPAP2C |
| FoxO signaling pathway | 0.010944751 | PRKAA2;SMAD3;GABARAP |
| Hepatitis C | 0.010944751 | SOCS3;IKBKG;TICAM1 |
| Insulin signaling pathway | 0.012324793 | SOCS3;PRKAA2;PDE3B |
| Hepatitis B | 0.01405576 | SMAD3;IKBKG;TICAM1 |
| Hippo signaling pathway | 0.015918287 | WNT10A;SMAD3;BBC3 |
| Caffeine metabolism | 0.017133087 | CYP1A2 |
| Pancreatic cancer | 0.021831228 | SMAD3;IKBKG |
| Influenza A | 0.022638742 | SOCS3;TRIM25;TICAM1 |
| Epithelial cell signaling in Helicobacter pylori infection | 0.023083063 | ATP6V1A;IKBKG |
| Drug metabolism - cytochrome P450 | 0.023719882 | CYP1A2;FMO3 |
| RIG-I-like receptor signaling pathway | 0.024363894 | TRIM25;IKBKG |
| Herpes simplex infection | 0.026130936 | SOCS3;IKBKG;TICAM1 |
| Chronic myeloid leukemia | 0.026338531 | SMAD3;IKBKG |
| Arrhythmogenic right ventricular cardiomyopathy (ARVC) | 0.02701076 | CACNG7;ITGA1 |
| Complement and coagulation cascades | 0.030474585 | F10;PLAUR |
| Proteoglycans in cancer | 0.033104713 | WNT10A;IGF2;PLAUR |
| Viral carcinogenesis | 0.033933856 | SCIN;HPN;IKBKG |
| Regulation of actin cytoskeleton | 0.037798185 | SCIN;ITGA1;LIMK1 |
| Dilated cardiomyopathy | 0.038670968 | CACNG7;ITGA1 |
| Morphine addiction | 0.039453604 | ADRBK1;PDE3B |
| Glycerophospholipid metabolism | 0.042643985 | PLA2G4B;PPAP2C |
| Glycosphingolipid biosynthesis - globo series | 0.047246693 | HEXA |
| Glucagon signaling pathway | 0.047603682 | PRKAA2;PDE3B |

**Supplementary Table 9:** Significant pathways for the case of the exclusive glioblastoma genes

| Pathways | P-value | Genes |
| --- | --- | --- |
| Antigen processing and presentation | 0.000180721 | HSPA6;CANX;TAP2;TAP1 |
| Cholinergic synapse | 0.000728521 | KCNQ1;CACNA1A;JAK2;GNAI2 |
| Toxoplasmosis | 0.000915733 | IRAK1;HSPA6;JAK2;GNAI2 |
| Protein processing in endoplasmic reticulum | 0.003404241 | HSPA6;CANX;DNAJC5G;MAN1A1 |
| Influenza A | 0.003855286 | HSPA6;EP300;FURIN;JAK2 |
| Tuberculosis | 0.004095319 | CEBPB;IRAK1;EP300;JAK2 |
| Herpes simplex infection | 0.004694332 | TAP2;TAP1;EP300;JAK2 |
| Primary immunodeficiency | 0.008058072 | TAP2;TAP1 |
| Axon guidance | 0.011258522 | SEMA4D;L1CAM;GNAI2 |
| ABC transporters | 0.011259419 | TAP2;TAP1 |
| Nucleotide excision repair | 0.012776092 | POLD2;DDB2 |
| Measles | 0.013529731 | IRAK1;HSPA6;JAK2 |
| Ubiquitin mediated proteolysis | 0.013797018 | RFWD2;WWP1;DDB2 |
| Phagosome | 0.018803532 | CANX;TAP2;TAP1 |
| Long-term depression | 0.020293029 | CACNA1A;GNAI2 |
| Long-term potentiation | 0.024250741 | RPS6KA3;EP300 |
| Renal cell carcinoma | 0.024250741 | EPAS1;EP300 |
| p53 signaling pathway | 0.026338531 | RFWD2;DDB2 |
| Leishmaniasis | 0.02923124 | IRAK1;JAK2 |
| Gastric acid secretion | 0.029973451 | KCNQ1;GNAI2 |
| Pertussis | 0.030723149 | IRAK1;GNAI2 |
| RNA degradation | 0.032244752 | LSM1;EXOSC2 |
| Synthesis and degradation of ketone bodies | 0.035914235 | OXCT1 |
| cAMP signaling pathway | 0.036316426 | EP300;CNGA4;GNAI2 |
| Taste transduction | 0.036982762 | CACNA1A;TAS1R3 |
| Epstein-Barr virus infection | 0.037702622 | IRAK1;HSPA6;EP300 |
| GABAergic synapse | 0.041122325 | CACNA1A;GNAI2 |
| Protein digestion and absorption | 0.042825019 | KCNE3;KCNQ1 |
| Morphine addiction | 0.043686176 | CACNA1A;GNAI2 |
| Progesterone-mediated oocyte maturation | 0.049892065 | RPS6KA3;GNAI2 |

**Supplementary Table 10:** Significant pathways for the case of breast cancer

| **Pathways** | **P-value** | **Genes** |
| --- | --- | --- |
| Signalling by NGF | 1.75E-10 | HDAC2;GSK3A;CDKN1B;HDAC1;SRC;ADCY4;PSEN2;PTEN;ADCY3;ADCY2;PIK3CB;PIK3R1;ADCY8;FOXO1;ADCY5;IKBKB;NRAS;APH1B;PPP2R1B;UBB;PPP2R1A;PRKAR2B;AKT2;CASP3;RPS6KA2;PRKAR2A;AKT3;AKT1;HRAS;NGFR;CHUK;STAT3;DUSP6;DNM1;NFKBIA;CREB1;PIK3CA;TRAF6;KRAS;SOS1;CALM2 |
| Cell Cycle, Mitotic | 1.57E-05 | CDKN1A;PSMD12;AHCTF1;PCNA;SEH1L;MCM8;ORC5L;UBE2D1;CDCA8;ANAPC10;CDC20;CCNB1;XPO1;TUBA1A;PPP2R1B;TUBB4;UBB;PPP2R1A;PRKAR2B;POLE;RFC5;PLK4;HSP90AA1;RFC4;NUP133;RFC1;UBE2C;PLK1;UBE2E1;RPA1;RPA2;KIF23;DYNLL1;CENPE;CCNA1;POLA1;CENPI;CDC16;CDK2;CENPL;KIF2C;CENPO;ANAPC2;MAD2L1 |
| Opioid Signalling | 6.38E-05 | ADCY4;ADCY3;ADCY2;ADCY8;GNG12;ADCY5;GNG13;GNA14;PPP3R1;GNG10;CREB1;GNGT2;GNG2;PPP2R1B;PPP2R1A;PRKAR2B;PRKAR2A;CALM2 |
| APC-Cdc20 mediated degradation of Nek2A | 8.15E-05 | CDC20;UBB;UBE2C;CDC16;UBE2E1;UBE2D1;ANAPC10;ANAPC2;MAD2L1 |
| Cell Cycle Checkpoints | 0.000281 | RFC5;CDKN1A;PSMD12;RFC4;UBE2C;MCM8;UBE2E1;ORC5L;RPA1;UBE2D1;RPA2;ANAPC10;CDC20;CCNB1;UBB;CDC16;CDK2;ATM;ANAPC2;MAD2L1 |
| Signaling by FGFR | 0.000386 | FGF5;FGF8;FGF9;FGF18;FGF20;FGFR4;FGF23;FGFR3 |
| Signaling by EGFR | 0.000806 | NRAS;PIK3CA;UBB;EGF;SRC;ADAM10;PTPN11;KRAS;PIK3R1;SOS1;HRAS |
| Hemostasis | 0.001169 | SRC;ITGB3;PIK3CB;PIK3R1;GNG10;NRAS;GNGT2;GNG2;FYN;STX4;HRAS;FGB;TGFB2;TGFB1;HSPA5;EGF;ITGA2;PRKCA;PTPN11;GNG12;GNG13;F7;F9;PIK3CA;KRAS;PFN1;SOS1;CALM2;PPIA;RAPGEF4 |
| Signaling by PDGF | 0.001924 | NRAS;PIK3CA;SRC;STAT3;PTPN11;KRAS;STAT6;PIK3CB;PIK3R1;SOS1;HRAS;NCK1 |
| HIV Infection | 0.004446 | PSMD12;SEH1L;NCBP1;CCNT1;NUP133;NCBP2;RNMT;PSIP1;AAAS;GTF2F1;NUP93;CD4;XPO1;UBB;NUP62;POLR2D;ERCC2;POLR2F;FYN;NUP88;WHSC2;CD247;PPIA;PAK2 |
| Signaling by Insulin receptor | 0.004894 | NRAS;PIK3CA;AKT2;KRAS;PIK3CB;PIK3R1;SOS1;HRAS;INS |
| mRNA Processing | 0.005089 | APOBEC1;NCBP1;NCBP2;RNMT;POLR2D;ERCC2;POLR2F;GTF2F1 |
| DNA Replication | 0.006983 | RFC5;CDKN1A;PSMD12;PCNA;RFC4;RFC1;MCM8;ORC5L;RPA1;RPA2;CCNA1;POLA1;UBB;CDK2;POLE |
| Axon guidance | 0.009378 | NRAS;CREB1;SRC;FYN;KRAS;SOS1;HRAS;SPTB |
| Cdc20:Phospho-APC/C mediated degradation of Cyclin A | 0.01262 | CDC20;CCNA1;PSMD12;UBB;UBE2C;CDC16;UBE2E1;UBE2D1;ANAPC10;ANAPC2;MAD2L1 |
| Regulation of beta-cell development | 0.016713 | AKT2;AKT3;AKT1;HNF1B;FOXO1;INS |
| Metabolism of nitric oxide | 0.018037 | HSP90AA1;NOS3;AKT1;CALM2 |
| DNA Repair | 0.021845 | RFC5;PCNA;RFC4;RFC1;MPG;RPA1;RPA2;POLB;APEX1;POLR2D;ERCC2;POLR2F;ATM;POLE |
| Phase 1 functionalization | 0.028126 | MAOB;MAOA;SMOX;PTGS2 |
| Signaling by TGF beta | 0.040992 | TGFB1;UBE2D1;TGFBR1;TGFBR2 |
| Apoptosis | 0.044336 | PSMD12;DIABLO;DYNLL1;ADD1;LMNB1;TJP1;PPP3R1;APC;UBB;CASP3;TNFSF10;PMAIP1;AKT1;BID;PAK2 |
| Botulinum neurotoxicity | 0.048485 | STX1B;SYT1;STX4;STX1A |
| Biological oxidations | 0.049583 | CYP2J2;MAOB;MAOA;SMOX;ACSM1;GLYAT;COMT;CYP46A1;CYP4F8;PTGS2;CYP8B1;ACSM2B;CYP24A1;NAT1;GSTA4 |

**Supplementary Table 11:** Significant pathways for the case of colon cancer

| Pathways | P-value | Genes |
| --- | --- | --- |
| Signalling by NGF | 3.89E-08 | HDAC2;HDAC3;CDKN1B;CLTA;ADCY3;ADCY8;MAPK8;NRAS;RAP1A;APH1B;PPP2R1B;UBB;PPP2R1A;PRKAR2B;AKT2;CASP3;RPS6KA2;AKT3;AKT1;THEM4;PLCG1;RAC1;PRKACA;HRAS;NGFR;NTRK2;CHUK;FRS2;RHOA;CREB1;TRAF6;MAPKAPK2;SOS1;CALM2 |
| Signaling by FGFR | 6.98E-06 | FGF5;FGF8;FGF9;FGF19;FGF18;FGF20;FGF23;FGFR3;FGF22;FGF4 |
| Cell Cycle, Mitotic | 0.000283 | CDKN1A;PCNA;CUL1;UBE2D1;CDC14A;POLD3;CCNB1;CDC23;XPO1;PPP2R1B;UBB;PPP2R1A;PRKAR2B;BUB3;FBXO5;BTRC;PRKACA;CLASP2;RFC5;PLK4;HSP90AA1;RFC3;RFC4;RFC1;PLK1;RPA1;DYNLL1;CCNA2;POLA1;POLA2;CDK4;RPA3;CENPC1;CDK2;CENPO;MCM2;MAD2L1 |
| DNA Repair | 0.000766 | RFC5;RFC3;PCNA;RFC4;RFC1;MPG;RPA1;BRCA1;FANCG;DDB1;POLD3;ERCC3;TDG;RPA3;ERCC2;ERCC6;MUTYH |
| Metabolism of nitric oxide | 0.002609 | HSP90AA1;NOS3;AKT1;NOSIP;CALM2 |
| DNA Replication | 0.002909 | RFC5;CDKN1A;RFC3;PCNA;RFC4;RFC1;RPA1;CCNA2;POLD3;POLA1;POLA2;UBB;RPA3;CDK2;MCM2 |
| Signaling by EGFR | 0.004059 | NRAS;UBB;EGF;GAB1;CLTA;SPRY2;PLCG1;SOS1;HRAS |
| Cell Cycle Checkpoints | 0.007489 | RFC5;CDKN1A;RFC3;RFC4;RPA1;UBE2D1;CCNB1;CDC23;UBB;RPA3;CDK2;BUB3;MCM2;MAD2L1;RAD9A |
| Telomere Maintenance | 0.007972 | RFC5;POLD3;POLA1;POLA2;RFC3;PCNA;RFC4;RFC1;RPA3;RPA1 |
| Opioid Signalling | 0.009196 | PPP3R1;GNG10;CREB1;PPP2R1B;PPP2R1A;PRKAR2B;ADCY3;ADCY8;GNG12;PRKACA;CALM2;PLCB2 |
| Signaling in Immune system | 0.014151 | ATF2;PROS1;ITGB2;PIK3R4;MAPK8;NRAS;IGKC;UBB;FYN;RIPK1;PLCG1;HRAS;CHUK;KIR3DL1;ATP1B2;LILRB4;MAP3K7IP1;CD4;IRF3;SLC7A8;TRAF6;UBE2N;PIK3C3;SOS1;PPIA;CFB |
| Axon guidance | 0.016531 | NRAS;CREB1;CNTN2;FYN;SOS1;HRAS;SPTB |
| APC-Cdc20 mediated degradation of Nek2A | 0.016621 | CDC23;UBB;UBE2D1;BUB3;MAD2L1 |
| Processing of Capped Intron-Containing Pre-mRNA | 0.018422 | SLBP;SF3A1;NCBP1;NCBP2;DHX9;DDX23;SFRS1;NXF1;PABPN1;HNRNPA2B1;SNRPG;CSTF1;SF3B1;SNRPA |
| Apoptosis | 0.021484 | DIABLO;GZMB;DYNLL1;PPP3R1;MAPK8;UBB;CASP6;CASP3;TNFSF10;AKT1;RIPK1;BMF;BID;PAK2;BCL2L1 |
| Signaling by TGF beta | 0.030474 | TGFB1;UBE2D1;TGFBR1;TGFBR2 |
| HIV Infection | 0.033506 | NCBP1;ELL;NCBP2;PSIP1;AAAS;NUP93;CD4;XPO1;ERCC3;UBB;NUP62;ERCC2;BANF1;FYN;NUP88;RAC1;BTRC;PPIA;PAK2 |

**Supplementary Table 12:** Significant pathways for the case of colorectal cancer

| Pathways | P-value | Genes |
| --- | --- | --- |
| Signalling by NGF | 7.06E-09 | GSK3A;CDKN1B;HDAC1;SRC;ADCY4;CLTA;PSEN1;ADCY8;APH1A;MAPK8;NRAS;RAP1A;APH1B;PPP2R1B;UBB;PPP2R1A;PRKAR2B;AKT2;CASP3;PRKAR2A;AKT3;AKT1;MAPK1;PLCG1;RAC1;HRAS;NGFR;FRS2;DUSP6;RHOA;CREB1;CAMK4;RAPGEF1;GRB2;TRIB3;SOS1;CALM2 |
| Signaling by FGFR | 1.21E-05 | FGF5;FGF17;KL;FGF8;FGF9;FGF19;FGF18;FGF20;FGF23;FGF22 |
| DNA Repair | 3.16E-05 | RFC5;RFC3;PCNA;RFC4;RFC1;MPG;H2AFX;RPA1;REV1;BRCA1;RAD23B;POLD3;SMUG1;ERCC3;ERCC4;ERCC1;TDG;POLE2;RPA3;ERCC2;ATM |
| DNA Replication | 0.000131 | RFC5;CDKN1A;RFC3;PCNA;RFC4;RFC1;MCM8;RPA1;CCNA2;POLD3;PSMA5;PSMB6;CCNA1;POLA1;POLA2;UBB;POLE2;RPA3;CDK2 |
| Telomere Maintenance | 0.000569 | RFC5;RFC3;ACD;PCNA;RFC4;RFC1;H2AFX;RPA1;POLD3;POLA1;POLA2;POLE2;RPA3 |
| Signaling by EGFR | 0.000619 | CDC42;NRAS;UBB;EGF;SRC;CLTA;MAPK1;GRB2;PLCG1;SOS1;HRAS |
| Opioid Signalling | 0.000998 | ADCY4;PLA2G4A;ADCY8;GNG12;GNG11;GNAO1;GNA15;GNG10;CREB1;PPP2R1B;PPP2R1A;PRKAR2B;PRKAR2A;CAMK4;CALM2 |
| Axon guidance | 0.002478 | NRAS;CREB1;SRC;MAPK1;FYN;GRB2;SOS1;HRAS;SPTB |
| Cell Cycle Checkpoints | 0.00272 | RFC5;CDKN1A;RFC3;RFC4;MCM8;UBE2E1;RPA1;UBE2D1;PSMA5;PSMB6;CCNB1;UBB;RPA3;CDK2;ATM;MAD2L1;RAD9A |
| HIV Infection | 0.002954 | NCBP1;ELL;NUP133;NCBP2;PSIP1;AAAS;PSMA5;PSMB6;CD4;XPO1;ERCC3;UBB;NUP85;NUP62;ERCC2;TCEB2;FYN;NUP88;CD247;RAC1;SLC25A5;BTRC;PPIA;PAK2 |
| Hemostasis | 0.005017 | CD63;SRC;PROS1;ITGB2;GNG10;NRAS;RAP1A;FYN;PLCG1;RAC1;HRAS;FGB;FCER1G;TGFB1;BRPF3;EGF;GNG12;F2;GNG11;F7;DOK2;SLC7A6;CD9;GRB2;SOS1;CALM2;PPIA |
| Cell Cycle, Mitotic | 0.00558 | CDKN1A;PCNA;MCM8;UBE2D1;POLD3;PSMB6;CCNB1;XPO1;PPP2R1B;UBB;PPP2R1A;PRKAR2B;NUP85;FBXO5;BTRC;RFC5;RFC3;RFC4;NUP133;RFC1;PLK1;UBE2E1;RPA1;DYNLL1;CCNA2;PSMA5;CCNA1;POLA1;POLA2;POLE2;RPA3;CDK2;CENPO;MAD2L1 |
| Phase 1 functionalization | 0.005994 | MAOB;MAOA;CYP1A1;PTGS2;PAOX |
| Signaling by PDGF | 0.026492 | NRAS;SRC;STAT1;RAPGEF1;MAPK1;GRB2;PLCG1;SOS1;HRAS |
| Processing of Capped Intron-Containing Pre-mRNA | 0.029683 | SF3B5;SLBP;SF3A1;SF3B3;NCBP1;NCBP2;DHX9;CSTF3;SFRS2;SFRS7;NXF1;SFRS4;CSTF1;SNRPA |
| Signaling by Insulin receptor | 0.031796 | NRAS;AKT2;MAPK1;GRB2;TRIB3;SOS1;HRAS |
| Apoptosis | 0.035028 | DIABLO;GZMB;DYNLL1;PSMA5;PSMB6;CASP7;MAPK8;UBB;CASP10;CASP3;TNFSF10;AKT1;FAS;BID;PAK2 |
| Signaling by TGF beta | 0.037137 | TGFB1;UBE2D1;TGFBR1;TGFBR2 |
| Signaling by Notch | 0.037137 | APH1A;NOTCH1;APH1B;PSEN1 |
| mRNA Processing | 0.038016 | APOBEC1;NCBP1;ERCC3;NCBP2;ERCC2;ADAR |
| Membrane Trafficking | 0.039248 | VAMP8;HSPA8;COPA;CLTA;ARRB1;VAMP2;DNM2 |
| Botulinum neurotoxicity | 0.043999 | SYT1;VAMP1;STX1A;VAMP2 |

**Supplementary Table 13:** Significant pathways for the case of rectum

| Pathways | P-value | Genes |
| --- | --- | --- |
| Signalling by NGF | 2.1E-11 | CDKN1B;HDAC1;SRC;ADCY4;ITPR1;PSEN1;ADCY8;ADCY7;CASP9;IKBKB;APH1A;SMPD2;NRAS;PPP2R1B;UBB;PPP2R1A;PRKAR2B;AKT2;CASP3;PRKAR2A;AKT3;AKT1;MAPK1;PLCG1;RAC1;HRAS;NGFR;PRKCI;FRS2;MAPK14;NFKBIA;ADAM17;ADORA2A;CAMK4;TRAF6;RAPGEF1;RAF1;CALM2 |
| Opioid Signalling | 0.000195 | ADCY4;ITPR1;ADCY8;GNG12;ADCY7;GNAO1;GNA14;GNG10;PPP2R1B;PPP2R1A;PRKAR2B;PRKAR2A;CAMK4;GNB3;CALM2 |
| DNA Repair | 0.000266 | RFC5;RFC3;PCNA;RFC4;MPG;RPA1;RPA2;BRCA1;MAD2L2;ERCC3;APEX1;ERCC1;TDG;RPA3;ERCC2;ATM;MUTYH |
| Signaling by EGFR | 0.000626 | ADAM17;NRAS;UBB;EGF;SRC;MAPK1;PLCG1;EPS15L1;RAF1;HRAS |
| DNA Replication | 0.001186 | RFC5;CDKN1A;RFC3;PCNA;RFC4;PRIM1;RPA1;RPA2;CDC6;CCNA2;POLA1;POLA2;UBB;RPA3;MCM4 |
| Signaling by FGFR | 0.002711 | FGF9;FGF19;FGF18;FGF20;FGF23;FGF22 |
| Cell Cycle Checkpoints | 0.003235 | RFC5;CDKN1A;RFC3;RFC4;RPA1;UBE2D1;RPA2;CDC6;CCNB1;UBB;RPA3;MCM4;ATM;MAD2L1;RAD9A |
| HIV Infection | 0.003638 | NCBP1;CCNT1;NCBP2;AAAS;GTF2F1;CD4;XPO1;ERCC3;UBB;ERCC2;AP2S1;TCEB2;FYN;NUP43;NUP88;RAC1;NUPL2;B2M;PPIA;PAK2;AP2M1 |
| Telomere Maintenance | 0.004248 | RFC5;POLA1;POLA2;RFC3;PCNA;RFC4;PRIM1;RPA3;RPA1;RPA2 |
| Axon guidance | 0.010412 | NRAS;SRC;MAPK1;FYN;RAF1;HRAS;SPTB |
| mRNA Processing | 0.019711 | APOBEC1;NCBP1;ERCC3;NCBP2;ERCC2;GTF2F1 |
| Cell Cycle, Mitotic | 0.026251 | CDKN1A;PCNA;PRIM1;UBE2D1;CCNB1;XPO1;PPP2R1B;UBB;PPP2R1A;PRKAR2B;RAD21;NUP43;RFC5;RFC3;RFC4;PLK1;RPA1;RPA2;CDC6;DYNLL1;CCNA2;POLA1;TUBB2C;POLA2;RPA3;MCM4;MAD2L1 |
| Signaling by PDGF | 0.027805 | NRAS;SRC;RAPGEF1;MAPK1;PLCG1;RAF1;HRAS;CRKL |
| Signaling by Insulin receptor | 0.042578 | NRAS;AKT2;MAPK1;RAF1;HRAS;INS |
| Synaptic Transmission | 0.04428 | GRIA2;DLG1;SYT1;MAOA;AP2S1;COMT;CACNG3;AP2M1;STX1A |
| Metabolism of nitric oxide | 0.045618 | NOS3;AKT1;CALM2 |
| Hemostasis | 0.047144 | CFD;TGFB1;ACTN2;EGF;SRC;ITPR1;ATP1B2;GNG12;GNG10;NRAS;PLCG2;GNB3;FYN;PLCG1;RAC1;ALDOA;TLN1;CALM2;PPIA;HRAS |

**Supplementary Table 14:** Significant pathways for the case of ovarian cancer

| Term | P-value | Genes |
| --- | --- | --- |
| Signalling by NGF | 2.54E-10 | HDAC2;CDKN1B;HDAC1;SRC;ADCY4;PIK3CB;PIK3R1;ADCY8;FOXO1;IKBKB;APH1A;SMPD2;PPP2CB;NRAS;APH1B;PPP2R1B;UBB;PPP2R1A;PRKAR2B;AKT2;CASP3;PRKAR2A;AKT3;RPS6KA1;AKT1;PLCG1;RAC1;HRAS;NGFR;MAP2K2;STAT3;FRS2;DUSP6;DNM1;NFKBIA;ADAM17;PIK3CA;TRAF6;GRB2;SOS1;CALM2 |
| Signaling by FGFR | 1.78E-05 | FGF8;FGF9;FGF19;FGF18;FGF20;FGFR4;FGF23;FGFR3;FGF4;FGF10 |
| Signaling by EGFR | 7.49E-05 | MAP2K2;EGF;SRC;ADAM10;PIK3R1;ADAM17;NRAS;PIK3CA;UBB;GRB2;PLCG1;SOS1;HRAS |
| DNA Replication | 0.000231 | RFC5;CDKN1A;RFC3;PCNA;RFC4;RPA1;RPA2;CDC6;CCNA2;CCNA1;POLA1;POLA2;UBB;PSMC1;PSMD2;RPA3;CDK2;PSMD3;PSMD1 |
| Signaling by Insulin receptor | 0.000519 | NRAS;MAP2K2;PIK3CA;RHEB;AKT2;GRB2;PIK3CB;PIK3R1;SOS1;HRAS;EIF4G1 |
| Cell Cycle Checkpoints | 0.001892 | RFC5;CDKN1A;RFC3;RFC4;RPA1;RPA2;CDC6;CCNB1;UBB;PSMC1;PSMD2;RPA3;CDK2;PSMD3;PSMD1;ATM;MAD1L1;MAD2L1 |
| Signaling by PDGF | 0.002121 | NRAS;MAP2K2;PIK3CA;SRC;STAT1;STAT3;GRB2;PIK3CB;PIK3R1;PLCG1;SOS1;HRAS |
| Signaling in Immune system | 0.003444 | ATF2;SRC;ITGB3;PROS1;SIRPG;PIK3CB;PIK3R1;LILRA1;IKBKB;NRAS;UBB;FYN;LBP;PLCG1;HRAS;MAP3K7;KIR2DS1;F2;HLA-G;LILRB4;NFKBIA;CD4;SLC7A8;PIK3CA;TRAF6;GRB2;CD226;SOS1;PPIA;CFB;NFKBIB |
| Hemostasis | 0.004968 | SRC;ITGB3;PROS1;SIRPG;PIK3CB;PIK3R1;NRAS;CFL1;FYN;PLCG1;RAC1;HRAS;TGFB1;HSPA5;SYK;ACTN2;EGF;GNG12;F2;GNG11;SLC7A8;PIK3CA;P2RX1;GNB3;GRB2;SOS1;CALM2;PPIA |
| DNA Repair | 0.005491 | RFC5;RFC3;PCNA;RFC4;MPG;H2AFX;RPA1;RPA2;RAD23B;POLB;SMUG1;APEX1;RPA3;ERCC2;ATM;MUTYH |
| Telomere Maintenance | 0.006443 | RFC5;POLA1;POLA2;RFC3;PCNA;RFC4;RPA3;H2AFX;RPA1;RPA2;HIST1H2BB |
| Cell Cycle, Mitotic | 0.006476 | CDKN1A;AHCTF1;PCNA;CENPA;CKS1B;PPP2CB;CCNB1;PPP2R1B;UBB;PPP2R1A;PRKAR2B;NUP85;PSMD2;PSMD3;PSMD1;RFC5;PLK4;RFC3;RFC4;PLK1;RPA1;RPA2;CDC6;DYNLL1;CCNA2;CCNA1;POLA1;NEDD1;POLA2;PSMC1;RPA3;CDK2;MAD1L1;PAFAH1B1;MAD2L1 |
| Axon guidance | 0.010022 | NRAS;MAP2K2;SRC;FYN;GRB2;SOS1;HRAS;SPTB |
| Apoptosis | 0.013875 | TRAF2;DYNLL1;H1F0;CASP7;UBB;CASP10;CASP3;PSMC1;PSMD2;PSMD3;TNFSF10;CTNNB1;CYCS;AKT1;PSMD1;BID;PAK2 |
| mRNA Processing | 0.016535 | APOBEC1;NCBP1;NCBP2;ERCC2;ADAR;GTF2F1;SUPT5H |
| HIV Infection | 0.017321 | NCBP1;NCBP2;PSIP1;AAAS;GTF2F1;SUPT5H;CD4;UBB;NUP85;PSMC1;PSMD2;ERCC2;PSMD3;TCEB2;PSMD1;BANF1;FYN;NUP88;RAC1;PPIA;PAK2;AP2M1 |
| Opioid Signalling | 0.019923 | PPP2CB;PPP2R1B;PPP2R1A;PRKAR2B;PRKAR2A;ADCY4;GNB3;PLA2G4A;ADCY8;GNG12;CALM2;GNG11 |
| Phase 1 functionalization | 0.029193 | MAOB;MAOA;PTGS2;PAOX |
| Gap junction trafficking and regulation | 0.035692 | GJA1;GJB1;SRC;GJA9;AP2M1;DNM1 |
| Signaling by Notch | 0.042492 | APH1A;NOTCH1;APH1B;ADAM10 |
| Signaling by Wnt | 0.047543 | PPP2CB;PPP2R1B;UBB;PPP2R1A;PSMC1;PSMD2;PSMD3;CTNNB1;PSMD1 |

**Supplementary Table 15:** Significant pathways for the case of glioblastoma

| Term | P-value | Genes |
| --- | --- | --- |
| Signalling by NGF | 5.54E-12 | HDAC3;CDKN1B;HDAC1;SRC;ADCY2;FURIN;PIK3CB;ADCY8;FOXO1;CASP9;IKBKB;PPP2CA;RPS6KA3;MAPK8;NRAS;RAP1A;PPP2R1B;IRAK1;UBB;PPP2R1A;PRKAR2B;AKT2;CASP3;PRKAR2A;AKT1;MAPK1;PLCG1;RAC1;HRAS;MAPK3;NGFR;NTRK2;MAP2K1;CHUK;STAT3;FRS2;MAPK14;DUSP6;DNM1;ADAM17;CREB1;PIK3CA;CAMK4;TRAF6;SOS1;CALM2 |
| Signaling by EGFR | 1.09E-05 | MAP2K1;EGF;SRC;ADAM10;PTPN11;ADAM17;NRAS;PIK3CA;UBB;MAPK1;SPRY1;PLCG1;SOS1;HRAS;MAPK3 |
| HIV Infection | 3.75E-05 | ARF1;CCNT1;PSIP1;AAAS;UBB;NUP85;NUP62;AP2S1;POLR2E;TCEB3;BANF1;NUP88;RAC1;B2M;PAK2;AP1M2;AP2M1;AP1M1;NCBP1;NUP133;APOBEC3G;NCBP2;XRCC5;TAF13;GTF2F1;CD4;CDK7;ERCC3;ERCC2;CD28;NUPL2;PPIA |
| Signaling in Immune system | 5.34E-05 | SRC;ITGB3;PROS1;KIR2DL1;PIK3CB;C3;IKBKB;MAPK8;NRAS;HLA-DMA;IRAK1;IGKC;UBB;ITGAX;MAPK1;RIPK1;PLCG1;B2M;HRAS;MAP3K7;HLA-DPA1;CHUK;KIR2DS1;PTPN11;L1CAM;HLA-G;ZAP70;CD4;IRF3;SLC7A8;PIK3CA;TRAF6;HLA-DRA;CD226;SOS1;PPIA;CFB;TLR3;NFKBIB |
| Opioid Signalling | 5.88E-05 | PLA2G4A;ADCY2;ADCY8;GNG12;GNG11;GNG13;GNAI2;GNAO1;PPP2CA;PPP3R1;GNG10;CREB1;PPP2R1B;PPP2R1A;PRKAR2B;PRKAR2A;CAMK4;CALM2;PLCB2 |
| DNA Repair | 0.000149 | RFC5;RFC3;PCNA;RFC4;XRCC5;RPA1;MRE11A;RPA2;BRCA1;BRCA2;DDB2;ALKBH3;CDK7;ERCC3;APEX1;RPA3;ERCC2;POLD2;POLR2E;ATM;MUTYH |
| Signaling by PDGF | 0.000153 | MAP2K1;SRC;STAT1;STAT3;FURIN;PTPN11;PIK3CB;CRKL;NRAS;PIK3CA;MAPK1;PLCG1;SOS1;HRAS;MAPK3 |
| Signaling by FGFR | 0.000636 | FGF17;KL;FGF8;FGF9;FGFR4;FGF23;FGFR3;FGF4 |
| Cell Cycle, Mitotic | 0.000691 | RB1;CDKN1A;PCNA;CUL1;UBE2D1;CASC5;CENPA;ANAPC10;PPP2CA;CCNB2;CCNB1;PPP2R1B;UBB;PPP2R1A;PRKAR2B;NUP85;POLD2;CDC45L;CCDC99;RFC5;CDT1;HSP90AA1;RFC3;RFC4;NUP133;PLK1;RPA1;RPA2;CDC6;DYNLL1;SMC1A;CDC25A;NDC80;CCNA1;POLA1;CDK7;POLA2;CCNE2;RPA3;CDC16;MAD1L1 |
| Cell Cycle Checkpoints | 0.000739 | RFC5;CDKN1A;RFC3;RFC4;RPA1;UBE2D1;RPA2;CDC6;ANAPC10;CDC25A;CCNB2;CCNB1;CCNE2;UBB;RPA3;CDC16;ATM;CDC45L;CLSPN;MAD1L1 |
| Axon guidance | 0.001634 | MAP2K1;NRAS;CREB1;SRC;MAPK1;SOS1;AGRN;HRAS;SPTB;MAPK3 |
| Synaptic Transmission | 0.001797 | SYT1;MAOA;COMT;SYN2;SLC6A3;SYN1;PANX1;PANX2;ALDH2;DLG4;AP2S1;GLUL;AP2M1;STX1A;VAMP2 |
| mRNA Processing | 0.002539 | APOBEC1;CDK7;NCBP1;ERCC3;NCBP2;ERCC2;POLR2E;ADAR;GTF2F1 |
| DNA Replication | 0.002881 | RFC5;RB1;CDT1;CDKN1A;RFC3;PCNA;RFC4;RPA1;RPA2;CDC6;CCNA1;POLA1;POLA2;UBB;RPA3;POLD2;CDC45L |
| Hemostasis | 0.003729 | SRC;ITGB3;PROS1;PIK3CB;RASGRP3;GNG10;NRAS;RAP1A;ITGAX;PLCG2;PLCG1;RAC1;TIMP1;HRAS;TGFB1;ACTN2;EGF;PTPN11;L1CAM;GNG12;GNG11;GNG13;F7;SLC7A8;PIK3CA;CD9;SOS1;CALM2;PPIA;PLCB2 |
| Signaling by Insulin receptor | 0.007899 | MAP2K1;NRAS;PIK3CA;AKT2;MAPK1;PIK3CB;SOS1;HRAS;MAPK3 |
| Apoptosis | 0.012868 | DYNLL1;ADD1;LMNB1;CASP9;PPP3R1;H1F0;MAPK8;UBB;CASP3;TNFSF10;CYCS;AKT1;FAS;RIPK1;CLSPN;BID;PAK2;HIST1H1B |
| Signaling by TGF beta | 0.014873 | TGFB1;UBE2D1;FURIN;TGFBR1;TGFBR2 |
| Metabolism of nitric oxide | 0.023066 | HSP90AA1;AKT1;NOSIP;CALM2 |
| Telomere Maintenance | 0.023721 | RFC5;POLA1;POLA2;RFC3;PCNA;RFC4;RPA3;POLD2;RPA1;RPA2 |
| Phase 1 functionalization | 0.035662 | PTGIS;MAOB;MAOA;PTGS2 |
| Gap junction trafficking and regulation | 0.046247 | GJA1;GJB1;SRC;GJA9;AP2M1;DNM1 |
| Mitochondrial Uncoupling Proteins | 0.049681 | UCP2;UCP1 |

**Supplementary Table 16:** Significant pathways for the case of glioma

| Term | P-value | Genes |
| --- | --- | --- |
| Signalling by NGF | 3.78E-10 | HDAC3;CDKN1B;HDAC1;ADCY2;PIK3CB;ADCY8;ADCY7;FOXO1;ADCY5;CASP9;IKBKB;PPP2CA;MAPK8;NRAS;RAP1A;PPP2R1B;UBB;PPP2R1A;PRKAR2B;AKT2;ADRBK1;CASP3;PRKAR2A;AKT1;MAPK1;PLCG1;RAC1;HRAS;MAPK3;NGFR;MAP2K1;CHUK;FRS2;MAPK14;DUSP6;ADAM17;CREB1;PIK3CA;TRAF6;GRB2;SOS1;CALM2 |
| Signaling by EGFR | 9.12E-06 | MAP2K1;EGF;ADAM10;PTPN11;ADAM17;NRAS;PIK3CA;UBB;MAPK1;GRB2;SPRY1;PLCG1;SOS1;HRAS;MAPK3 |
| Opioid Signalling | 4.83E-05 | PLA2G4A;ADCY2;ADCY8;GNG12;ADCY7;GNG11;ADCY5;GNG13;PPP2CA;PPP3R1;GNG10;CREB1;PPP2R1B;PPP2R1A;PRKAR2B;ADRBK1;PRKAR2A;CALM2;PLCB2 |
| HIV Infection | 6.46E-05 | ARF1;CCNT1;PSIP1;AAAS;UBB;NUP85;NUP62;AP2S1;POLR2E;TCEB2;BANF1;NUP88;RAC1;B2M;PAK2;AP1M2;AP1M1;NCBP1;NUP133;NCBP2;XRCC5;TAF13;HMGA1;GTF2F1;CD4;CDK7;ERCC3;ERCC2;CD28;PPIA;NUP37 |
| Signaling by Insulin receptor | 0.000232 | MAP2K1;NRAS;EEF2K;PIK3CA;AKT2;PDE3B;MAPK1;GRB2;PIK3CB;SOS1;HRAS;MAPK3 |
| Cell Cycle Checkpoints | 0.000252 | RFC5;CDKN1A;RFC3;RFC4;RPA1;UBE2D1;RPA2;CDC6;ANAPC10;CDC25A;CCNB2;CCNB1;CCNE2;UBB;RPA3;CDC16;CDK2;ATM;CDC45L;CLSPN;MAD1L1 |
| DNA Repair | 0.00031 | RFC5;RFC3;PCNA;RFC4;XRCC5;RPA1;MRE11A;RPA2;BRCA1;BRCA2;ALKBH3;CDK7;ERCC3;APEX1;ERCC1;RPA3;ERCC2;POLR2E;ATM;MUTYH |
| Signaling by FGFR | 0.000578 | FGF17;KL;FGF8;FGF9;FGF23;FGFR3;FGF22;FGF4 |
| Signaling in Immune system | 0.000645 | PROS1;KIR2DL1;PIK3CB;C3;IKBKB;MAPK8;NRAS;HLA-DMA;IGKC;UBB;ITGAX;MAPK1;IKBKG;PLCG1;B2M;HRAS;MAP3K7;HLA-DPA1;FCER1G;CHUK;PTPN11;TICAM1;HLA-G;ZAP70;CD4;IRF3;PIK3CA;TRAF6;HLA-DRA;GRB2;CD226;SOS1;PPIA;TLR3;NFKBIB |
| Cell Cycle, Mitotic | 0.000939 | RB1;CDKN1A;PCNA;CUL1;UBE2D1;CENPA;ANAPC10;PPP2CA;CCNB2;CCNB1;PPP2R1B;UBB;PPP2R1A;PRKAR2B;NUP85;CDC45L;CCDC99;RFC5;CDT1;HSP90AA1;RFC3;RFC4;NUP133;PLK1;RPA1;RPA2;CDC6;DYNLL1;SMC1A;CDC25A;NDC80;POLA1;CDK7;POLA2;CCNE2;RPA3;CDC16;CDK2;MAD1L1;NUP37 |
| Signaling by PDGF | 0.001158 | MAP2K1;STAT1;PTPN11;PIK3CB;CRKL;NRAS;PIK3CA;MAPK1;GRB2;PLCG1;SOS1;HRAS;MAPK3 |
| Axon guidance | 0.001466 | MAP2K1;NRAS;CREB1;MAPK1;GRB2;SOS1;AGRN;HRAS;SPTB;MAPK3 |
| mRNA Processing | 0.002301 | APOBEC1;CDK7;NCBP1;ERCC3;NCBP2;ERCC2;POLR2E;ADAR;GTF2F1 |
| Synaptic Transmission | 0.003852 | MAOA;COMT;SYN2;SLC6A3;SYN1;PANX1;PANX2;DLG1;ALDH2;DLG4;AP2S1;GLUL;STX1A;VAMP2 |
| Metabolism of nitric oxide | 0.005071 | HSP90AA1;NOS3;AKT1;NOSIP;CALM2 |
| Hemostasis | 0.005442 | PROS1;PIK3CB;RASGRP3;GNG10;NRAS;RAP1A;ITGAX;PLCG1;RAC1;HRAS;FCER1G;TGFB1;HSPA5;SYK;F10;ACTN2;EGF;PTPN11;GNG12;GNG11;GNG13;APBB1IP;PIK3CA;CD9;GRB2;SOS1;CALM2;PPIA;PLCB2 |
| DNA Replication | 0.005551 | RFC5;RB1;CDT1;CDKN1A;RFC3;PCNA;RFC4;RPA1;RPA2;CDC6;POLA1;POLA2;UBB;RPA3;CDK2;CDC45L |
| Apoptosis | 0.011201 | DYNLL1;ADD1;LMNB1;BBC3;CASP9;TJP1;PPP3R1;H1F0;MAPK8;UBB;CASP3;TNFSF10;CYCS;AKT1;FAS;CLSPN;BID;PAK2 |
| Signaling by TGF beta | 0.014042 | SMAD3;TGFB1;UBE2D1;TGFBR1;TGFBR2 |
| Inhibition of HSL | 0.03054 | AKT2;PDE3B |
| Phase 1 functionalization | 0.034074 | MAOA;CYP1A2;FMO3;PTGS2 |
| Telomere Maintenance | 0.04779 | RFC5;POLA1;POLA2;RFC3;PCNA;RFC4;RPA3;RPA1;RPA2 |
| Mitochondrial Uncoupling Proteins | 0.048398 | UCP2;UCP1 |

**Supplementary Table 17:** Common and Exclusive mechanisms between the seven different cancer types

| **Cancer Types** | **Number of Common Pathways** | **Common Pathways** |
| --- | --- | --- |
| **BreastCancer,, ColonCancer, ColorectalCancer,, Glioblastoma, Glioma, OvarianCancer,, Rectum** | 10 | Axon guidance, Cell Cycle Checkpoints, Signaling by FGFR, DNA Repair, DNA Replication, Opioid Signalling, HIV Infection, Cell Cycle, Mitotic Signalling by NGF, Signaling by EGFR |
| **BreastCancer, ColonCancer, ColorectalCancer, Glioblastoma, Glioma, OvarianCancer** | 1 | Apoptosis |
| **BreastCancer, ColorectalCancer, Glioblastoma, Glioma, OvarianCancer, Rectum** | 4 | Hemostasis Signaling by PDGF mRNA Processing Signaling by Insulin receptor |
| **ColonCancer, ColorectalCancer, Glioblastoma, Glioma, OvarianCancer, Rectum** | 1 | Telomere Maintenance |
| **BreastCancer, ColonCancer, ColorectalCancer, Glioblastoma, Glioma** | 1 | Signaling by TGF beta |
| **BreastCancer, ColonCancer, Glioblastoma, Glioma, Rectum** | 1 | Metabolism of nitric oxide |
| **BreastCancer, ColorectalCancer, Glioblastoma, Glioma, OvarianCancer** | 1 | Phase 1 functionalization |
| **ColonCancer, Glioblastoma, Glioma, OvarianCancer** | 1 | Signaling in Immune system |
| **Glioblastoma, Glioma, Rectum** | 1 | Synaptic Transmission |
| **BreastCancer, ColonCancer** | 1 | APC-Cdc20 mediated degradation of Nek2A |
| **BreastCancer, ColorectalCancer** | 1 | Botulinum neurotoxicity |
| **ColonCancer, ColorectalCancer** | 1 | Processing of Capped Intron-Containing Pre-mRNA |
| **ColorectalCancer, OvarianCancer** | 1 | Signaling by Notch |
| **Glioblastoma, OvarianCancer** | 1 | Gap junction trafficking and regulation |
| **Glioblastoma, Glioma** | 1 | Mitochondrial Uncoupling Proteins |
| **BreastCancer** | 3 | Cdc20:Phospho-APC/C mediated degradation of Cyclin A, Regulation of beta-cell development, Biological oxidations |
| **ColorectalCancer** | 1 | Membrane Trafficking |
| **OvarianCancer** | 1 | Signaling by Wnt |
| **Glioma** | 1 | Inhibition of HSL |

**Supplementary Table 18:** Common and exclusive repurposed drugs of each cancer type

| **Cancer Types** | **Number of Common Drugs** | **Common Drugs** |
| --- | --- | --- |
| **BreastCancer, ColorectalCancer, OvarianCancer, Rectum** | 1 | idarubicin |
| **ColonCancer, ColorectalCancer, OvarianCancer, Rectum** | 2 | fulvestrant, amsacrine |
| **ColonCancer, Glioblastoma, Glioma, Rectum** | 2 | trichostatin-a, vorinostat |
| **ColorectalCancer, Glioma, OvarianCancer, Rectum** | 1 | 7-Ethyl-10-hydroxycamptothecin |
| **BreastCancer, Glioblastoma, Glioma** | 1 | palbociclib |
| **ColonCancer, ColorectalCancer, Rectum** | 1 | diphenyleneiodonium |
| **ColonCancer, ColorectalCancer, Glioma** | 1 | wortmannin |
| **ColonCancer, Glioma, Rectum** | 1 | Fedratinib |
| **ColorectalCancer, OvarianCancer, Rectum** | 1 | teniposide |
| **BreastCancer, OvarianCancer** | 1 | 1-(4-Chlorobenzyl)-4-(4-phenyl-1-piperazinyl)-1H-pyrazolo[3,4-d]pyrimidine |
| **ColonCancer, ColorectalCancer** | 2 | fluorouracil, N-[3-(1,3-Benzothiazol-2-yl)-5,6,7,8-tetrahydro-4H-thieno[2,3-c]azepin-2-yl]acetamide |
| **ColonCancer, Rectum** | 2 | ellipticine, N-[3-(1,3-Benzothiazol-2-yl)-5,6-dihydro-4H-thieno[2,3-c]pyrrol-2-yl]acetamide |
| **ColorectalCancer, OvarianCancer** | 2 | naproxol, neratinib |
| **ColorectalCancer, Glioma** | 1 | 2-Methyl-4-(5-methyl-[1,3,4]thiadiazol-2-ylsulfanyl)-quinoline |
| **OvarianCancer, Rectum** | 1 | 5-[(Benzyloxy)methyl]-7-(1-pyrrolidinylmethyl)-8-quinolinol |
| **Glioblastoma, Rectum** | 2 | simvastatin, geldanamycin |
| **Glioblastoma, Glioma** | 6 | devazepide, PHA-793887, 4-[6-(Adamantan-1-yl)-7-hydroxy-2-naphthyl]benzoic, acid, 5H-quino[8,7-c][1,2]benzothiazine, 6,6-dioxide, XMD11-50, BI-2536 |
| **BreastCancer** | 17 | ruxolitinib, rottlerin, (2R)-3-Cyclopentyl-2-[4-(methylsulfonyl)phenyl]-N-(1,3-thiazol-2-yl)propanamide, N-[(4-Chlorophenyl)(2-methyl-1H-indol-3-yl)methyl]-4-methyl-2-pyridinamine, 3,5-Dichloro-2-hydroxy-N-(4-methoxy-3-biphenylyl)benzenesulfonamide, (2E)-2-Cyano-3-(3,5-di-tert-butyl-4-hydroxyphenyl)prop-2-enethioamide, N-(2-Aminophenyl)-4-{[({(2S,3S)-10-[(cyclohexylcarbonyl)amino]-5-[(2R)-1-hydroxy-2-propanyl]-3-methyl-6-oxo-3,4,5,6-tetrahydro-2H-1,5-benzoxazocin-2-yl}methyl)(methyl)amino]methyl}benzamide, triamterene, entinostat, N-[(5-Bromo-8-hydroxy-7-quinolinyl)(2-thienyl)methyl]acetamide, camptothecin, [(3-Chlorophenyl)hydrazono]malononitrile, {[4-(Trifluoromethoxy)phenyl]hydrazono}malononitrile, 4-{2-[(6-Chloro-4-quinazolinyl)amino]ethyl}phenol, (5E)-5-{[5-(1H-Benzimidazol-2-ylsulfanyl)-2-furyl]methylene}-3-isopropyl-1,3-thiazolidine-2,4-dione, 3-(3-Benzoyl-6-chloro-4,5-dihydroxy-1-benzofuran-7-yl)-2,4-pentanedione, (2E,2'E)-N,N'-(Methylenedi-4,1-cyclohexanediyl)bis[2-cyano-3-(3,4-dihydroxyphenyl)acrylamide] |
| **ColonCancer** | 9 | aminopurvalanol-a, (5E)-5-(6-Quinoxalinylmethylene)-1,3-thiazolidine-2,4-dione, (3Z)-5-(3-Pyridinyl)-3-(1H-pyrrol-2-ylmethylene)-1,3-dihydro-2H-indol-2-one, purvalanol-a, selumetinib, gestrinone, N-methyl-N-(3-((2-(2-oxo-2,3-dihydro-1H-indol-5-ylamino)-5-trifluoromethyl-pyrimidin-4-ylamino)-methyl)-pyridin-2-yl)-methanesulfonamide, rhodomyrtoxin-b, [6-Hydroxy-2-(4-hydroxyphenyl)-1-benzothiophen-3-yl][4-(4-isopropyl-1-piperazinyl)phenyl]methanone |
| **ColorectalCancer** | 8 | 2-Methyl-6-[(E)-2-phenylvinyl]pyridine, 2-Chloro-N-[2-(cyclohexylamino)-2-oxo-1-(3-pyridinyl)ethyl]-N-(4-fluorophenyl)acetamide, 4-[(4E)-4-{[5-(4,5-Dimethyl-2-nitrophenyl)-2-furyl]methylene}-3-methyl-5-oxo-4,5-dihydro-1H-pyrazol-1-yl]benzoic, acid, fluticasone, maprotiline, emodic-acid, irinotecan, clofarabine |
| **Rectum** | 6 | N-[(5-Chloro-8-hydroxy-7-quinolinyl)(cyclopropyl)methyl]acetamide, mitoxantrone, 3,6-Dimethyl-9-[6-(2-oxiranyl)-6-oxohexyl]decahydropyrrolo[1,2-a][1,4,7,10]tetraazacyclododecine-1,4,7,10-tetrone, N-[3-(1,3-Benzothiazol-2-yl)-5-isopropyl-5,6-dihydro-4H-thieno[2,3-c]pyrrol-2-yl]acetamide, N'-[(E)-(3-Allyl-2-hydroxyphenyl)methylene]-2-(4-benzyl-1-piperazinyl)acetohydrazide, thioisobutyric, acid, S-(6-(4-phenyl-2-thiazolylcarbamoyl)hexyl), ester |
| **OvarianCancer** | 10 | daunorubicin, K784-3131, etoposide, SIB-1893, N-[(5-Fluoro-8-hydroxy-7-quinolinyl)(2-thienyl)methyl]acetamide, AS-605240, hinokitiol, PD-0325901, malonoben, PD-184352 |
| **Glioblastoma** | 9 | MG-132, lylamine, paclitaxel, JAK3-inhibitor-VI, NVP-TAE684, retinyl, fluvastatine, 2-Chloro-N-(3-chloro-4-methoxyphenyl)-N-{2-oxo-2-[(2-phenylethyl)amino]-1-(2-thienyl)ethyl}acetamide, (5S,11aR)-2-Butyl-5-(4-methoxyphenyl)-5,6,11,11a-tetrahydro-1H-imidazo[1',5':1,6]pyrido[3,4-b]indole-1,3(2H)-dione |
| **Glioma** | 7 | 4-[(1-Methyl-1H-tetrazol-5-yl)sulfanyl]-5-[4-(trifluoromethyl)phenyl]thieno[2,3-d]pyrimidine, 5-(4-Bromophenyl)-4-[(1-methyl-1H-tetrazol-5-yl)sulfanyl]thieno[2,3-d]pyrimidine, brazilin, ingenol, thapsigargin, 2-Methyl-2-propanyl, [4-(3-{[7-(hydroxyamino)-7-oxoheptyl]carbamoyl}-1,2-oxazol-5-yl)phenyl]carbamate, GW-405,833 |
